# ERF transcription factor StPti5 is a regulator of endophyte community maintenance in potato

**DOI:** 10.1111/nph.71240

**Published:** 2026-05-24

**Authors:** Tjaša Lukan, Karmen Pogačar, Barbara Kraigher, Katja Stare, Teja Grubar Kovačič, Maja Zagorščak, Marko Petek, Polonca Stefanic, Anže Vozelj, Valentina Levak, Tjaša Mahkovec Povalej, Juan M. García, Maria J. Pozo, Eva Álvarez, José M. Franco‐Zorrilla, Maja Križnik, Špela Baebler, Ines Mandić‐Mulec, Kristina Gruden

**Affiliations:** ^1^ Department of Biotechnology and Systems Biology National Institute of Biology Ljubljana 1000 Slovenia; ^2^ Jožef Stefan International Postgraduate School Jamova cesta 39 Ljubljana 1000 Slovenia; ^3^ Chair of Microbial Ecology and Physiology, Department of Microbiology Biotechnical Faculty, University of Ljubljana Ljubljana 1000 Slovenia; ^4^ Department of Soil and Plant Microbiology Estación Experimental del Zaidín (CSIC) Granada 18008 Spain; ^5^ Department of Plant Molecular Genetics Centro Nacional de Biotecnología‐CSIC Darwin 3 Madrid 28049 Spain

**Keywords:** arbuscular mycorrhizal fungus *Rhizophagus irregularis*, *Bacillus subtilis*, endophyte colonization mechanism, ERF transcription factor StPti5, microbial‐associated molecular pattern (MAMP)‐triggered immunity, *Solanum tuberosum*

## Abstract

- We have recently identified an ethylene response factor, StPti5, as a susceptibility factor that negatively regulates immune responses to diverse pathogens.
- Here, we investigated the role of StPti5 in the processes involved in the colonization of potato with beneficial organisms. RNA‐seq showed that at the time of *Bacillus subtilis* biofilm establishment, immune responses in interacting roots were attenuated, and a complex transcriptional network was triggered, with ethylene signaling being a central module and *StPti5* strongly induced. Interestingly, the response is intensified if plants are inoculated by two antagonistic *B. subtilis* strains.
- While StPti5 is not involved in the establishment of biofilm on roots, we show that bacterial abundance increases in shoots of *StPti5*‐silenced plants. Remarkably, root colonization by the arbuscular mycorrhizal fungus *Rhizophagus irregularis* was also higher in the *StPti5*‐silenced plants. To decipher the mechanistic basis of StPti5 function, we performed a DAP‐seq experiment and showed that StRIN13, a regulator of plant immune signaling, is a direct target of StPti5.
- StPti5 is involved both in suppressing defense against harmful and limiting colonization by beneficial microbes. Such a mechanistic understanding of plant–microbe interaction paves the way for sustainable crop management.

## Introduction

Plant–microbe and microbe–microbe interactions in the rhizosphere determine plant health and productivity (Mauchline & Malone, 2017). Beneficial microorganisms are common in rhizosphere soils. Some can endophytically colonize plants, frequently improving plant nutrition and plant growth, and protect plants from disease and abiotic stresses through a wide variety of mechanisms (Timmusk *et al*., 2017; Pozo *et al*., 2021). They can be efficient and environmentally friendly alternatives to chemical pesticides and fertilizers, due to their multiple benefits for the plants and their long shelf life, which is comparable to that of agrochemicals (Kandel *et al*., 2017). However, the current limitation for a wider adoption of microbial inoculants in agriculture is the reproducibility of the results under field conditions (O'Callaghan *et al*., 2022). Additional knowledge, such as a deeper understanding of the mechanisms involved in plant colonization and maintenance of the plant microbiome, is required to improve the use of microbes as plant protectants (Lutz *et al*., 2023).

The plant immune system is central to shaping the plant response to beneficial and pathogenic microorganisms, regulating the extension and successful colonization of plant tissues by mutualistic organisms and preventing the invasion by parasitic ones. Recognition of pathogens leads to the activation of interconnected plant hormonal signaling pathways, mediating downstream transcriptional reprogramming, resulting in effective defense responses (Baebler *et al*., 2014; Ngou *et al*., 2022). When a plant interacts with beneficial microbes, microbial‐associated molecular pattern (MAMP)‐triggered immunity (MTI) is activated similarly to interaction with pathogens (Tsai *et al*., 2023). Beneficial microbes develop several strategies to overcome this immune response and colonize the plant. One strategy to evade immune recognition is to alter microbe‐associated molecular patterns, such as flagellin. Alternatively, microbes develop tolerance to toxic compounds produced, for example, reactive oxidative species (ROS), or they actively suppress MTI (Teixeira *et al*., 2021). They can also modify the metabolism of the plant to allow for easier penetration, for example, suppressing the synthesis of suberin (Verbon *et al*., 2023).

Successful colonization by beneficial microbes can induce resistance to pathogenic bacteria and herbivores. Mechanisms of induced resistance (IR) are not well understood, but it has been shown to involve jasmonic acid (JA), salicylic acid (SA), and ethylene signaling pathways (Pieterse *et al*., 2014; De Kesel *et al*., 2021; Yu *et al*., 2022). Several individual transcription factors have also been shown to be important for establishing the IR. As described for bacteria, root colonization by beneficial fungi can also boost plant immunity. For example, root colonization by arbuscular mycorrhizal fungi (AMF) is tightly regulated, and the modulation of immune responses during the symbiosis establishment can also lead to IR in systemic tissues (Fiorilli *et al*., 2024). Therefore, understanding the fine‐tuned regulation of plant immune responses during colonization by beneficials is essential for optimizing bioinoculant‐based strategies in agriculture, allowing their establishment and promoting their benefits for plant health.

Bacterial social behavior plays a crucial role in the successful colonization of plant tissues. Bacteria often exist in multicellular groups called biofilms (Flemming *et al*., 2016), where they engage in competition for resources but also in cooperative (synergistic) interactions that enhance community productivity (Mitri & Richard, 2013). These social engagements are most often mediated through secreted molecules that are believed to have a profound impact on bacterial diversity, spatial organization, and ecological functions (Flemming *et al*., 2016; Asfahl & Schuster, 2017). *Bacillus subtilis* is capable of different forms of social interactions besides biofilm formation, such as quorum sensing (QS), group motility (swarming), and kin discrimination (Kalamara *et al*., 2018). Non‐kin *B. subtilis* strains form a striking boundary between approaching swarms (Stefanic *et al*., 2015; Lyons *et al*., 2016), exclude a non‐kin competitor from a common swarm (Kraigher *et al*., 2022), and do not mix on *Arabidopsis thaliana* roots, while kin strains merge on solid medium and cohabit root surfaces (Stefanic *et al*., 2015). *Bacillus subtilis* strains also produce a range of antibiotics, surfactants, secondary metabolites, and bioactive volatiles expected to modulate social interactions between bacteria and with the plant (Stefanic *et al*., 2015; Lareen *et al*., 2016; Lyons *et al*., 2016; Liu *et al*., 2024).

The *B. subtilis* QS system comprises the signaling peptide ComX, which positively regulates the synthesis of surfactin (encoded by srf operon), one of the most potent surfactants and a lipopeptide antibiotic (Oslizlo *et al*., 2014; Kalamara *et al*., 2018). Surfactin acts as a signaling molecule during biofilm formation, a promoter of horizontal gene transfer, and as an inducer of physiological changes in plants during colonization with bacteria (Ongena *et al*., 2007; López *et al*., 2009; Chaudhry *et al*., 2021; Danevčič *et al*., 2021; Rahman *et al*., 2021). Despite evidence for *in situ* QS‐dependent expression of surfactin, the link between QS and surfactin on plant roots and how QS affects plant colonization and plant immune response is less understood.

As the potato (*Solanum tuberosum*), the third most important crop in the world, is exceptionally sensitive to a wide range of environmental stresses (Vilvert *et al*., 2022), studying its interaction with beneficial microbes is crucial to ensure an efficient, environmentally friendly system of plant protection based on modulation of its microbiome, which impacts plant growth and health (Song *et al*., 2025). The aim of this study was to investigate the mechanisms underlying the interaction between potato and beneficial microbes. Different *B. subtilis* strains and mutants were used to investigate the principles of bacterial social interactions and their interaction with the plant. We hypothesized that both potato root and shoot tissues respond to interaction with diverse *B. subtilis* strains at the gene expression level and that this response is independent of the bacterial strain or potato genotype. We next hypothesized that the intensity of the plant response is influenced by both the extent of bacterial colonization and the interaction between bacteria when the plant is inoculated with different strains.

We identified the transcription factor StPti5 as a potential regulatory hub and hypothesized that it plays a role in the colonization of potato by microbes. Using transgenic plants with silenced *StPti5*, we show that *B. subtilis* abundance is increased in shoots of plants with silenced *StPti5*, suggesting that StPti5 maintains optimal *B. subtilis* abundance in colonized plants. Interestingly, a similar function was also confirmed for the establishment of the mutualistic symbiosis between potato and AMF. We also show that StPti5 regulates immune signaling by activation of StRIN13.

## Materials and Methods

### Plant materials

Potato (*Solanum tuberosum* L.) cv Rywal, a transgenic line expressing *NahG* and thus impaired in SA accumulation (NahG‐Rywal; Baebler *et al*., 2014), *StPti5*‐silenced NahG‐Rywal transgenic line (shPti5‐NahG‐Rywal; Coll *et al*., 2024), and cv Désirée were used in this study. Plants were grown in stem node tissue culture or in soil as described in Supporting Information Methods S1.

### Plant transformation

*StPti5*‐silenced Rywal transgenic lines were prepared by introducing the short hairpin RNA (shRNA) construct pH7GWIWG2_shPti5 (Coll *et al*., 2024) into Rywal plants using *Agrobacterium tumefaciens* strain LBA4404 (Lukan *et al*., 2018). The transformed bacteria were used to perform stable transformation of cv Rywal, using stem internodes from *in vitro* plantlets as described in Lukan *et al*. (2023), grown on appropriate selection media. Two transgenic lines, shPti5 L2 and L6, were used in subsequent experiments and grown in the same conditions as the other genotypes.

### *Bacillus subtilis* strains

Wild‐type (WT) *B. subtilis* strains PS‐216, PS‐218, and PS‐68 (Stefanic & Mandic‐Mulec, 2009) tagged with red fluorescent protein (mKate2) or yellow fluorescent protein (YFP), and PS‐216 mutants with impaired surfactin production or impaired QS were used in our experiments. For the detailed genotypes, see Methods S2.

### *Bacillus subtilis* IAA production detection

The production of indole‐3‐acetic acid (IAA) by *B. subtilis* strains was assessed following the protocol of Pramanik *et al*. (2023), see Methods S3.

### Plant inoculations

On day one, *B. subtilis* from a permanent culture was plated on solid LB medium with the appropriate antibiotic (100 μg ml^−1^ spectinomycin or 5 μg ml^−1^ chloramphenicol) and incubated overnight at 37°C. On day two, a single colony was resuspended in 3 ml of LB medium with antibiotics and grown overnight at 200 rpm and 37°C. On day three at noon, 30 μl of the bacterial culture was inoculated in 3 ml of LB medium without antibiotics and grown for 3 h at 200 rpm at 37°C (OD_600_ between 0.08 and 0.2). Bacterial culture was then diluted in 3 ml of MS30 to an OD_600_ of 0.02 (*c*. 10^7^ CFU ml^−1^), pipetted into a 12‐well plate, and incubated for 16 h at 22°C on a low‐speed shaker at 11 rpm.

On day four, after a 16‐h incubation, at 10^8^–10^9^ CFU ml^−1^, potato plants were added to the bacterial culture. The potato shoots were separated from the bacterial culture using a UV‐sterilized parafilm cover (Fig. S1a–c). In all experiments, except those studying the role of QS and surfactin, bacterial cultures were vortexed before adding them to the plants. For the study of interactions, cultures of different strains were mixed at a 1 : 1 ratio, or mixed with MS30 media to obtain control monocultures, to achieve the same final CFU ml^−1^ as in the strain interaction studies. Plants were incubated from 15 min to 26 h at 11 rpm in a growth chamber before sampling. Details of individual experiments are given in Methods S4.

### Mycorrhizal symbiosis establishment and quantification

The mycorrhizal inoculum was maintained as described in Chabot *et al*. (1992). Potato plantlets growing in 125‐ml pots with a mixture of soil, sand, and vermiculite (3 : 2 : 1, v : v : v) were inoculated by adding a 1‐cm piece of the monoxenic culture, containing *c*. 50 *Rhizophagus irregularis* spores, fungal hyphae, and colonized carrot roots. For the non‐mycorrhizal controls, a piece of medium containing only uninfected carrot roots was applied. Plants were grown in a growth chamber (day : night cycle, 16 h, 24°C : 8 h, 19°C; relative humidity: 50%). The experiment was repeated twice, and eight independent plants per genotype were analyzed.

Mycorrhizal colonization of roots and establishment of symbiosis were evaluated histochemically and molecularly at 4 wk post‐inoculation, as described in García *et al*. (2020), and Giovannetti & Mosse (1980). See Methods S5 for details.

### Flg22 treatment

Flagellin 22 (Eurogentec; AS‐62633) (Flg22) was prepared as a 1‐μM solution (Sano *et al*., 2014) in MS30 medium. Two‐week‐old potato plants from tissue culture were transferred to 12‐well microtiter plates with Flg22 suspension and MS30 medium as a control. Root samples were collected 2 h after the start of incubation, and leaf samples were collected 26 h after the start of incubation. All samples were immediately frozen in liquid nitrogen and stored at −80°C for further analysis.

### Confocal microscopy

A Stellaris 8 confocal microscope with HC PL FLUOTAR 10×/0.30 DRY objective (Leica Microsystems, Wetzlar, Germany) was used to detect emission of YFP and mKate2 on the roots of plants in the experiment for investigating timing of biofilm formation, *B. subtilis* morphology after internalization in plants, biofilm formation pattern when studying bacteria–plant social interactions, biofilm presence on the roots of plants sampled for RNA‐seq, biofilm formation in transgenic plants with silenced *StPti5* and prior all quantitative polymerase chain reaction (qPCR) experiments to confirm roots colonization. A Stellaris 5 confocal microscope with HC PL APO CS2 20×/0.75 DRY objective (Leica Microsystems) was used to detect emission of YFP in the roots of plants in the experiment for internalization of bacteria. A Leica TCS LSI confocal macroscope with Plan APO 5× objective (Leica Microsystems) was used to detect emission of YFP when investigating biofilm formation on the roots of mutants with attenuated surfactin production (see Methods S6 for settings).

### Transmission electron microscopy

Samples were prepared for examination with transmission electron microscopy (TEM) using the negative staining method. Potato leaves were infiltrated with *B. subtilis* PS‐216 YFP (10^7^ CFU ml^−1^), grown in liquid MS30 and sampled 3 d post‐infiltration. Homogenized leaves with infiltrated bacteria or bacteria from liquid culture were applied to the grids and stained with 1% (w/v) water solution of uranyl acetate (see Methods S7 for experimental details). The grids were observed by TEM TALOS L120C (Thermo Fisher Scientific, Waltham, MA, USA), operating at 100 kV, and representative micrographs were acquired (camera Ceta 16 M) using velox software.

### qPCR analysis

The shoots and elongation zones of roots from *B. subtilis*‐inoculated and non‐inoculated potato plants (see description in the ‘Plant inoculations’ in the ‘Materials and Methods’ section) were sampled by snap freezing in liquid nitrogen in four or five biological replicates (plants). Samples were homogenized, RNA was isolated, DNase‐treated, and quality‐controlled, and then reverse‐transcribed. To determine *B. subtilis* abundance in systemic shoot tissue, samples were homogenized, and genomic DNA was isolated (see Methods S8 for details).

Gene expression of potato marker genes and abundance of microorganisms in potato samples were analyzed by qPCR (see Table S1 for a full list of genes and their descriptions). The expression of nine potato marker genes (*StRBOHD*, *StHSP70*, *StPti5*, *St13‐LOX*, *StPR1B*, *StACO*, *StBGLU2*, *StCPI8*, *StCAB*) involved in different steps of immune signaling was determined and normalized to the expression of two validated reference genes, *StCOX* and *StEF‐1*, as described previously (Petek *et al*., 2014). Relative copy numbers of *B. subtilis BsComQ* or *BsGyrB* were determined and normalized to those of a single‐copy gene, StNPR1. The standard curve method was used for relative gene expression quantification using quantGenius (http://quantgenius.nib.si) (Baebler *et al*., 2017).

For molecular assessment of mycorrhizal colonization, the expression of the *RiEF* gene and the markers of symbiotic functionality, *RiMST2* and *StPT4*, were determined in roots by qPCR using gene‐specific primers. Relative quantification of specific mRNA levels was performed using the comparative ΔΔCt method (Livak & Schmittgen, 2001), using *StEF‐1α* as a reference.

### Statistical analysis of qPCR, microbial abundance and imaging data

Statistical analysis of qPCR and microbial data was conducted in R v.4.4.1 (R Core Team, 2024). All details of statistical analysis are given in Methods S9.

### RNA‐seq analysis

Two independent experiments were performed for the RNA‐seq analysis to study potato response to *B. subtilis*. For a detailed description of potato growth and *B. subtilis* inoculation, see ‘Plant inoculations’ in the ‘Materials and Methods’ section. In addition, an independent RNA‐seq analysis was performed to identify downstream targets of StPti5. Details of RNA‐seq experimental design are given in Methods S10.

Differential gene expression analysis was performed in R v.3.6.1 using the limma package v.3.40.6 (Ritchie *et al*., 2015). See Methods S3 for details. Genes with Benjamini‐Hochberg FDR‐adjusted *P*‐values < 0.05 and |log_2_FC| > 1 were considered significantly differentially expressed. RNA‐seq results tables were supplemented with UniTato geneIDs (Zagorščak *et al*., 2024) and Mercator4 v.6 functional annotations (Schwacke *et al*., 2019). Venn diagrams were plotted using a modified limma::vennDiagram function. Raw and normalized read counts were deposited in the GEO under accession nos. GSE232028 and GSE313881.

Gene Set Enrichment Analysis was performed with the GSEA Desktop application v.4.1.0 (Subramanian *et al*., 2005), using the MapMan ontology‐based gene sets (Ramšak *et al*., 2021). See Methods S10 for details.

### Network analysis

A Mechanistic Plant Stress Signaling (PSS) (https://skm.nib.si/) (Bleker *et al*., 2024) reaction network was pre‐processed using DiNAR's (Zagorščak *et al*., 2018) pre‐processing app. Interactions among different molecular entities (such as genes, proteins, or transcripts) are defined on a functional cluster level, mostly encompassing *A. thaliana* gene identifiers. Therefore, potato gene identifiers (Petek *et al*., 2020) were first translated to Arabidopsis gene identifiers using a blast+ reciprocal best hit search (Camacho *et al*., 2009) on proteomes (TranslationTables section in the https://github.com/NIB‐SI/DiNAR/ repository). The top three potato identifiers for each Arabidopsis identifier were considered and further prioritized to the functional cluster using DiNARs' prioritization script. Experimental data were superimposed onto the PSS network and visualized using DiNAR. Separate condition‐specific networks, as defined by differential expression contrasts, were exported as individual interactive .html files for further examination.

### Protein expression and DNA affinity purification

StPti5 coding sequence was fused to maltose‐binding protein in pDEST‐TH1 through LR cloning. Expression of recombinant protein and DNA affinity purification (DAP‐seq) experiments were performed as described (López‐Vidriero *et al*., 2021), see Methods S11 for details.

Genes in which DAP‐seq revealed StPti5 binding to GCC box (GCCGCC sequence, (Coll *et al*., 2024)) in the peak of at least 200 bp positioned in the region between −500 and +100 bp from the transcription start site (TSS) were marked as positive, as it was shown in large‐scale DAP‐seq studies that ethylene response factors (ERFs) most frequently bind close to the TSS (Baumgart *et al*., 2025). Expression of these genes was checked in *StPti5*‐silenced plants infected with the virus, as it was shown that the StPti5 protein accumulates in infected cells but not in normally grown leaves (Coll *et al*., 2024). The gene was considered a target of StPti5 if identified after filtering of DAP‐seq results and if the difference in expression in the viral infection zone of shPti5 plants and non‐transgenic plants was at least 0.8 on the log_2_ scale (|log_2_FC| > 0.8), and they were significantly regulated in at least one genotype following viral inoculation. Next, the identified targets of StPti5 were assigned their function using MapMan4 ontology via mercator4 v.7 (Schwacke *et al*., 2019) and their interactions with other proteins using the STRING database v.12.0 search (https://string‐db.org/, Szklarczyk *et al*., 2023).

## Results

### Stable *B. subtilis* biofilm formation on potato roots coincides with transcriptional responses in plant roots

**Fig. 1:**
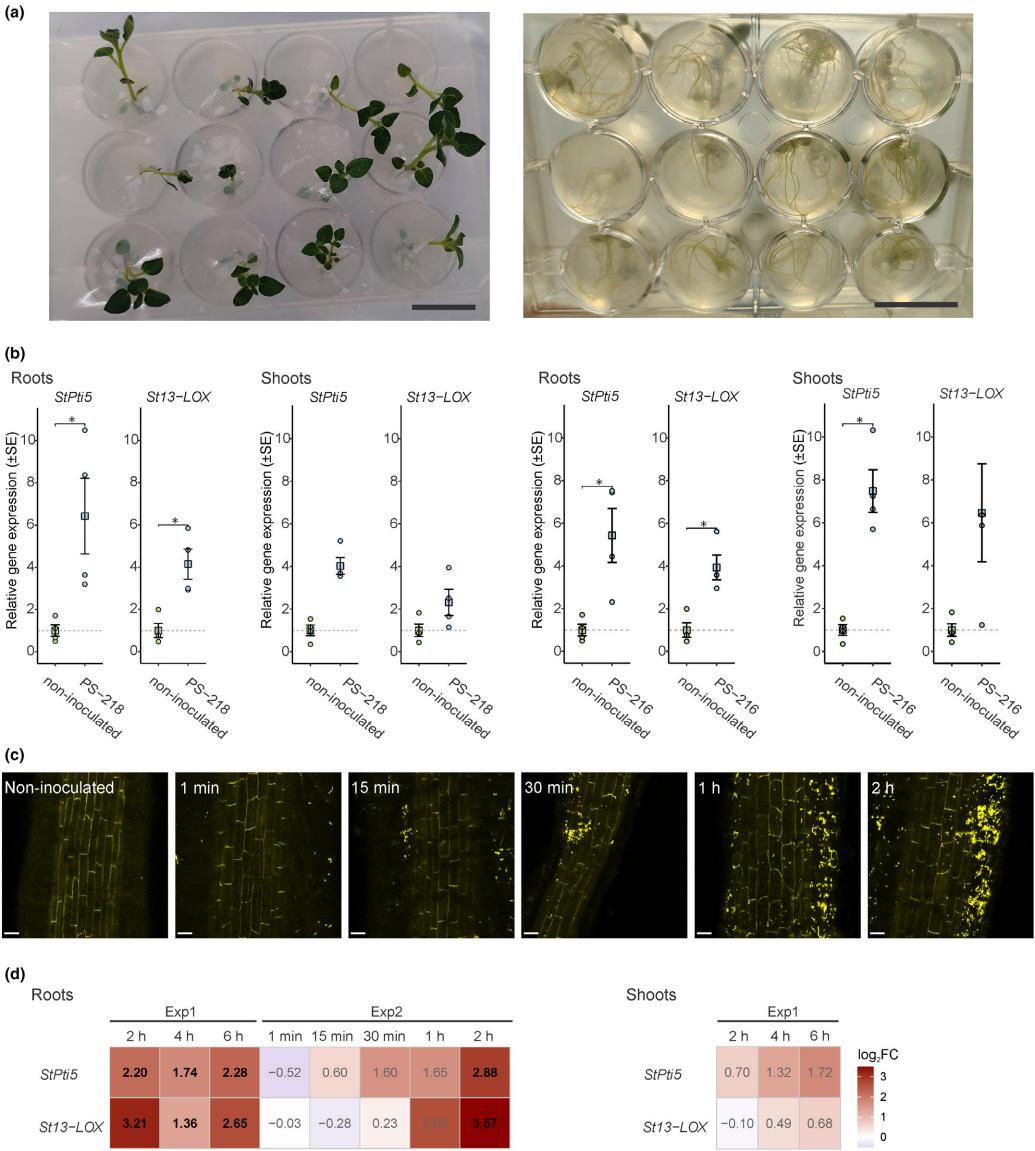
Dynamics of biofilm formation and plant responses of potato to *Bacillus subtilis*. (a) The experimental setup. The potato shoots (left) were separated from the bacterial culture using parafilm cover, while the roots (right) were immersed in the bacterial culture. Bars, 2 cm. (b) Potato response to bacterial colonization, measured by expression of *StPti5* and *St13‐LOX*. Relative expression of the target genes in roots and shoots of *B. subtilis* PS‐218 and PS‐216 YFP inoculated (10^7^ CFU ml^−1^) and non‐inoculated plants, after overnight incubation is shown. Permutational *t*‐test was used to determine differences between treatments (*n* = 4). *P*‐values were adjusted using the Benjamini‐Hochberg (BH) procedure. For visualization purposes, relative gene expression was scaled to the average gene expression of the non‐inoculated group. Individual measurements (circles), mean (squares), and SE are shown. Asterisks denote a statistically significant difference (*P*‐value < 0.05). Additional data is given in Supporting Information Table S2 and Fig. S4. (c) Biofilm formation (YFP fluorescence) on potato roots after incubation with *B. subtilis* PS‐218 YFP of high density (10^8^–10^9^ CFU ml^−1^) for a specified time. Bars, 50 μm. (d) Dynamics of *StPti5* and *St13‐LOX* expression in roots and shoots of potato plants after inoculation with *B. subtilis* strain PS‐218 culture of high density (10^8^–10^9^ CFU ml^−1^) and incubation for a specified time. Up‐ (shaded in red) or downregulation (shaded in blue) was determined by comparing expression in inoculated and non‐inoculated plants, represented as log_2_FC. Bold‐formatted log_2_FC values denote statistically significant regulation, as determined using permutational *t*‐test with BH adjustment. Exp: experiment. Additional data is available in Tables S5 (Exp1) and S6 (Exp2). Biofilm formation and gene expression data were similar for all tested *B. subtilis* strains and potato genotypes (Tables S3, S4).

We here studied the response of potato to colonization by different *B. subtilis* strains (PS‐218, PS‐216 and PS‐68). We first studied external root colonization and biofilm formation. All three strains formed a biofilm on potato roots (Figs S2, S3). We next selected eight genes known to be involved in the potato plant immune response (Lukan *et al*., 2020; Coll *et al*., 2024) as potential indicators of potato MTI to *B. subtilis* colonization and followed their expression in potato roots and shoot tissue after overnight inoculation with bacteria. The genes showed diverse expression profiles, some of which were tissue‐specific (Tables S2, S3). Two genes, an ERF *StPti5*, which requires an active ethylene pathway for activation and mediates crosstalk of ethylene and SA pathways (Coll *et al*., 2024) and a gene involved in JA synthesis, *St13‐LOX* (Lukan *et al*., 2020) were reproducibly upregulated in roots and in shoots (Figs 1a,b, S4; Tables S2, S3). The results were similar for all three tested *B. subtilis* strains and both potato genotypes, cv Rywal and unrelated cv Désirée (Tables S2–S4; Fig. S4).

To study the dynamics of biofilm formation, we set up an experiment using a high‐density bacterial inoculum (10^8^–10^9^ CFU ml^−1^). In such a setup, biofilm reproducibly began forming after the first 15 min of incubation, and it was consistently present over the entire root surface after a 2‐h incubation period (Fig. 1c). Interestingly, the induction of *StPti5* and *St13‐LOX* genes was also detected when the biofilm was formed uniformly on the root (Fig. 1d; Tables S5, S6). All further experiments were conducted using high‐density bacterial inoculum to reproducibly capture the first responses of the plant to bacterial root colonization.

### *Bacillus subtilis* colonization triggers a complex regulatory network in potato

**Fig. 2:**
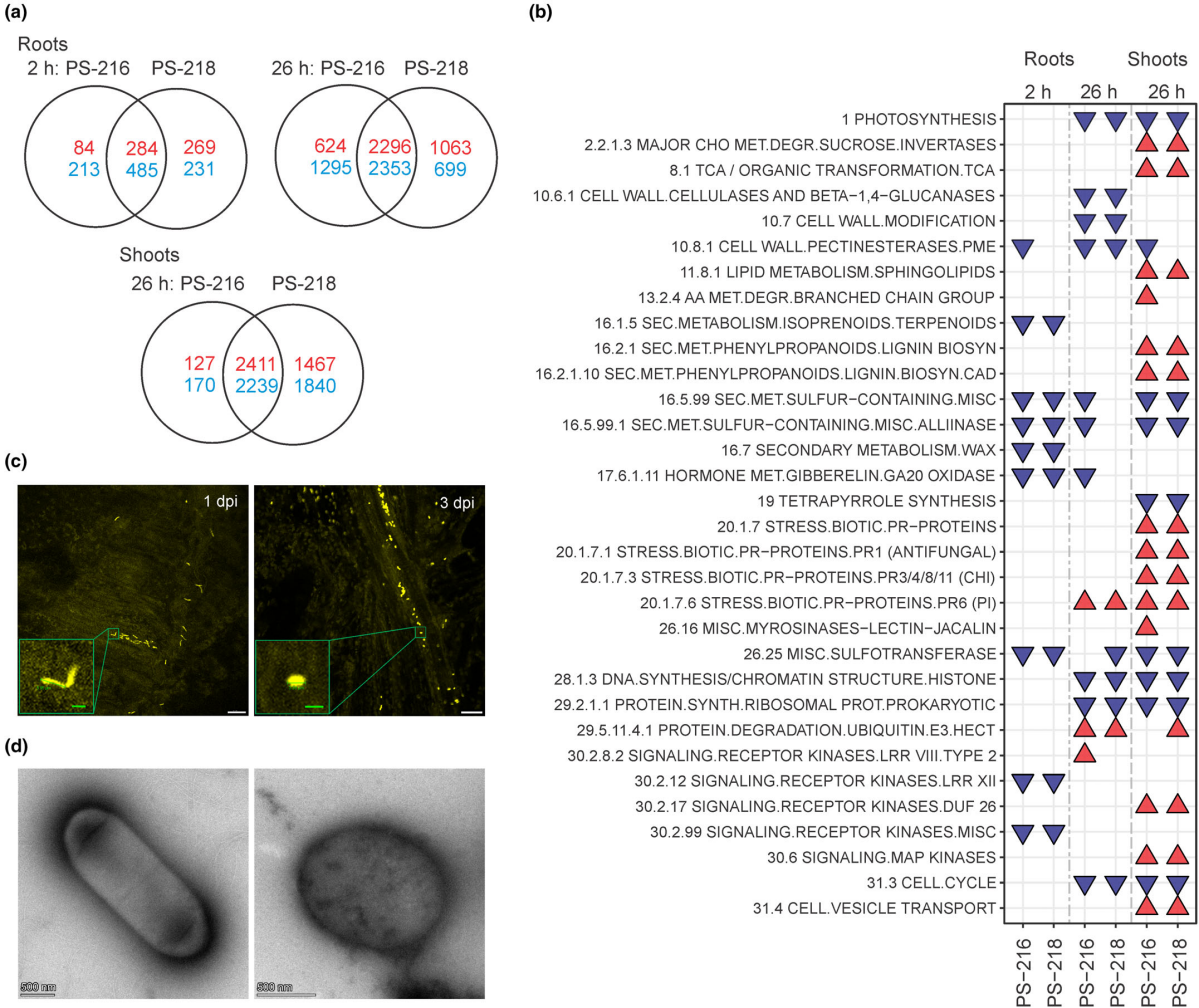
Potato adjusts to colonization with *Bacillus subtilis* by repression of immune signaling and cell wall synthesis. (a) Numbers of unique and common differentially expressed genes in plant roots and shoots after 2‐ and 26‐h incubation with *B. subtilis* PS‐216 and PS‐218 (10^8^–10^9^ CFU ml^−1^) as determined by RNA‐seq (Supporting Information Table S7A). Blue: downregulated genes, red: upregulated genes. Confirmation of results by qPCR is given in Table S9. (b) Visualization of results in the context of biological pathways. For each comparison between *B. subtilis*‐inoculated and non‐inoculated plants (in columns for each strain and time point), gene sets enriched for up‐ or downregulated genes (*q*‐value < 0.1) are shown and designated as red triangles or blue triangles for shoots and roots at different time points. MapMan ontology BINs were used to generate gene sets. PS‐216: PS‐216 YFP, PS‐218: PS‐218 mKate2. See Table S8 for all results. (c) *B. subtilis* morphology is changed after internalization in the plant stem. *Bacillus subtilis* morphology in plant stem 1 dpi (day post‐inoculation, left) and 3 dpi (right) changed to a more rounded form. See Fig. S7 for more data. Bars, 25 μm in the main images and 3 μm in the inset images. (d) Electron micrograph of free‐living (left) and *in planta* (in leaves) living *B. subtilis* PS‐216 (right). Bars, 500 nm.

To investigate the plant response to *B. subtilis* colonization in more detail, we next performed RNA‐seq analysis of colonized roots and systemic shoot tissue. Our special interest was in the initial stage of colonization, as we intended to capture plant responses related to initial *B. subtilis* recognition. Therefore, the root transcriptome was analyzed at the time of biofilm formation (2 hpi) and 24 h later (26 hpi). The response of the plant was stronger at the later time point (Figs 2a, S5; Table S7A). Moreover, to explore potential activation of MTI and/or IR, the shoot transcriptome was analyzed at the later time point. For both root and shoot tissues, there was a substantial overlap in differentially expressed genes among plants inoculated with two different strains (Figs 2a, S5).

Many processes related to the primary line of defense were downregulated in roots at the time of biofilm formation, including diverse immune receptors, genes involved in cell wall modifications, and in the synthesis of terpenoids and waxes (Fig. 2b; Table S8). Interestingly, among the genes that were upregulated in roots already at the time of biofilm formation were, in the majority, genes coding for transcription factors, hormonal synthesis, signaling components, and components of protein degradation. Several ERF transcription factors were upregulated in addition to the already identified *StPti5*, as well as many MYB, WRKY, bZIP, and NAC transcription factor coding genes (Table S7B). We also identified several lipoxygenases (JA synthesis) upregulated, as well as genes involved in ethylene and gibberellin synthesis, and in Ca^2+^ signaling (Table S7B). The response of most of these genes was intensified at the later time point in both roots and shoots (Table S7B).

Remarkably, more induced gene sets were detected in shoots. Receptor kinases were upregulated, as were MAP kinases and executors of plant defense, such as beta‐glucanases, alpha amylase inhibitors, *PR1* proteins, and *PR5* protein encoding genes (Fig. 2b; Tables S7B, S8). Among these genes associated with transcriptional regulation in IR, the SA receptor *NPR1/3/4* genes were upregulated only in shoots (Table S7B).

We next followed the spread of *B. subtilis* through the plant and bacterial colonization of internal compartments. Bacterial internalization from the biofilm begins through the roots, as we detected endophytic bacteria in root parenchyma 1 dpi (Fig. S6). We also detected bacteria on the stem as early as 1 dpi, both on the surface and internalized (Fig. S7). Interestingly, while the plant responds to *B. subtilis* colonization, *B. subtilis* also adapts once internalized, as its morphology starts to change to a more rounded form as early as 2 dpi (Figs 2c, S7). Using TEM, we observed that most free‐living bacteria are multiplying (75%), while multiplication was attenuated in *in planta* living bacteria (17% of bacteria are dividing; Figs 2d, S8, Zenodo (doi: 10.5281/zenodo.18174134)).

### Biofilm formation and plant responses are attenuated in potato roots inoculated with *B. subtilis* quorum‐sensing and surfactin‐deficient mutants

**Fig. 3:**
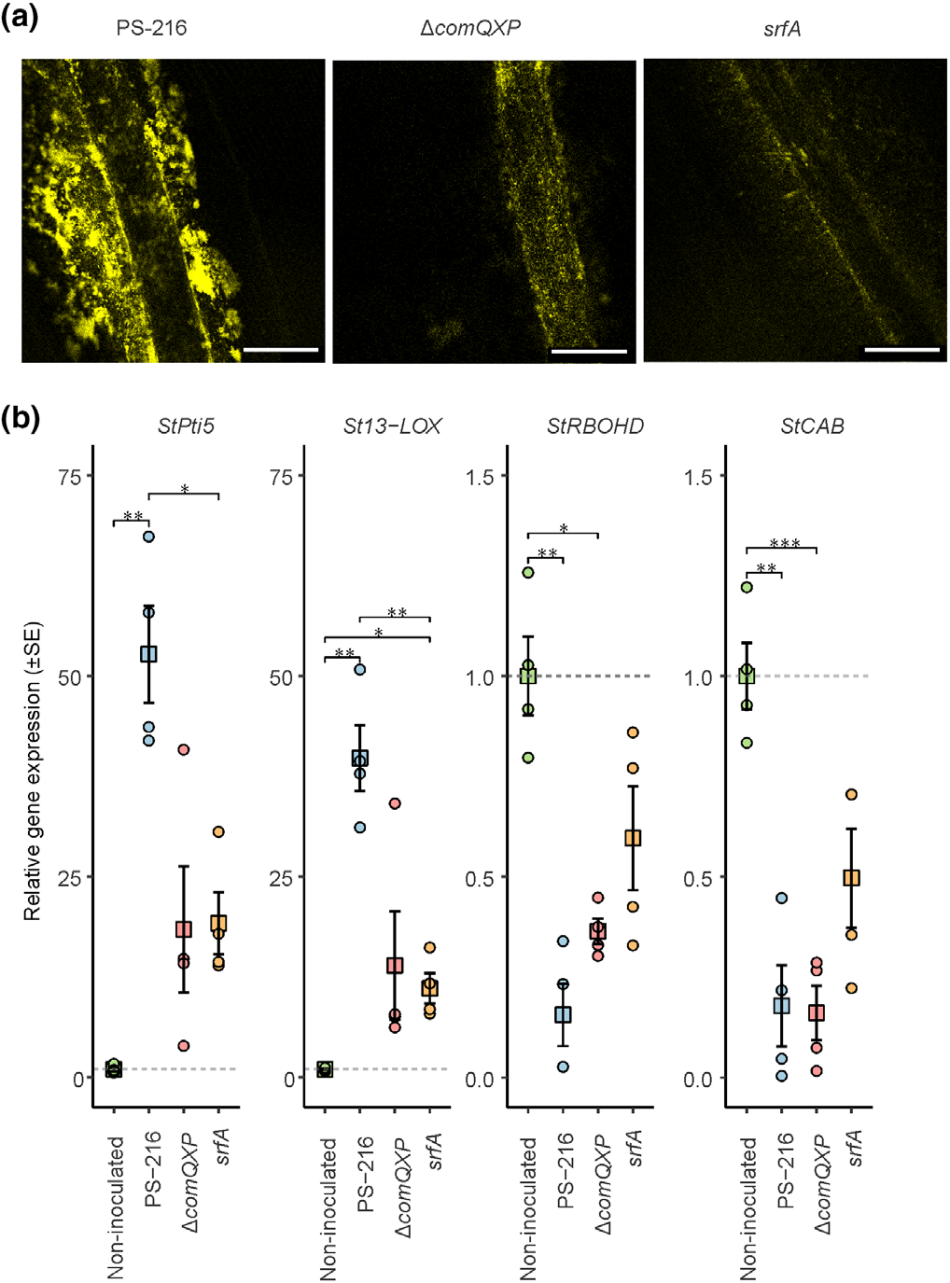
Induction of plant responses is dependent on efficient *Bacillus subtilis* biofilm formation on roots. (a) Biofilm formation on potato roots after 26‐h incubation with YFP‐labeled *B. subtilis* PS‐216 (left), quorum‐sensing‐negative *B. subtilis* YFP‐labeled mutant PS‐216 ΔcomQXP (middle), and PS‐216 *srfA* mutant (right) culture (10^8^–10^9^ CFU ml^−1^). Bars, 500 μm. (b) Expression of selected genes (*StPti5*, *St13‐LOX*, *StRBOHD*, *StCAB*) in non‐inoculated potato roots and after 26‐h incubation with *B. subtilis* PS‐216 YFP, PS‐216 Δ*comQXP* YFP and PS‐216 *srfA* YFP culture (10^8^–10^9^ CFU ml^−1^). Games Howell post‐hoc test was used to determine differences between groups (*n* = 4). For visualization purposes, relative gene expression was scaled to the average gene expression of the non‐inoculated group. Individual measurements (circles), mean (squares), and SE are shown. Asterisks denote statistically significant difference (*P*‐value < 0.05 for * and < 0.01 for **). See Supporting Information Table S11 for the results of all genes for both roots and shoots.

To better understand the mechanisms that trigger plant responses, we examined the role of *B. subtilis* ComX peptide‐mediated QS and surfactin production in potato–*B. subtilis* interaction. We used two PS‐216 mutant strains, one with deletion of the *comQXP* gene cluster, encoding for the genes involved in QS, which also regulate surfactin production (PS‐216 Δc*omQXP*) (Oslizlo *et al*., 2014), and another with a mutation in the *srfA* operon, which is unable to produce surfactin (PS‐216 *srfA*). Both mutants are reported to still form biofilms in liquid media (Thérien *et al*., 2020). The biofilm formed by both mutants on potato roots was barely detectable, while strong biofilm formation was formed by WT bacteria (Figs 3a, S9; Table S10). The reduced biofilm‐forming ability was reflected in the potato response to *B. subtilis*. The induction of JA synthesis and ethylene signaling markers observed in response to the WT bacteria was attenuated in the plant response to the mutant strains. Similarly, the colonization‐associated downregulation of apoplastic ROS generator *StRBOHD* and photosynthesis marker *StCAB* was less pronounced in plants colonized by the mutant strains (Fig. 3b; Table S11).

To confirm that biofilm establishment is required for successful activation of plant responses and that *B. subtilis* secondary metabolites are not sufficient, we treated plants with conditioned media in which bacteria were growing. Results showed that conditioned media alone did not trigger any response in treated plants, despite containing surfactin (Fig. S10; Table S12). Therefore, we conclude that *B. subtilis* biofilm formation on the roots is required for the induction of potato response.

### Non‐kin social interactions of *B. subtilis* strains on potato roots intensify potato immune response

**Fig. 4:**
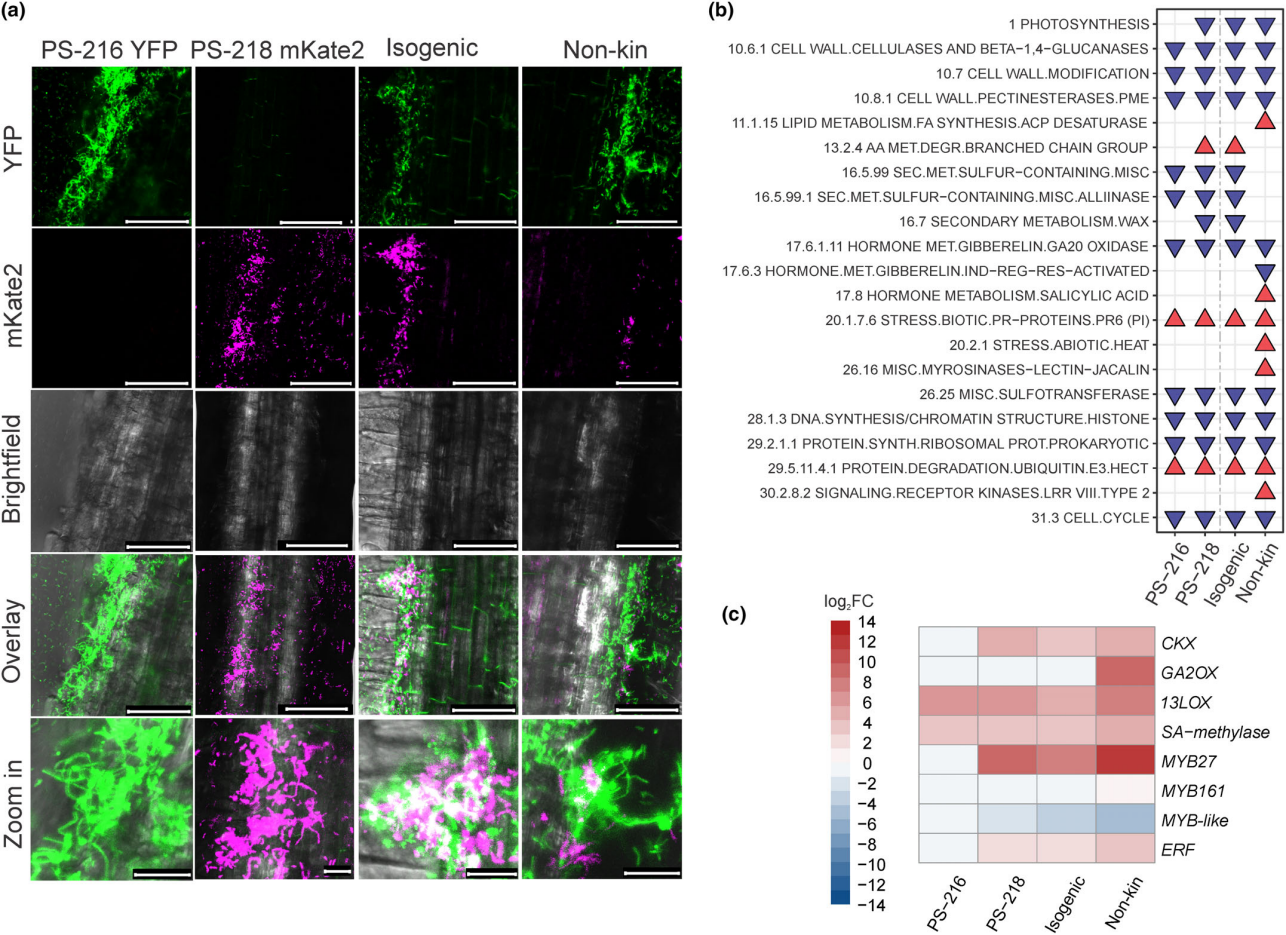
Social interactions of *Bacillus subtilis* strains on potato roots intensify potato immune response. (a) Colonization of potato roots by isogenic and non‐kin *B. subtilis* strains (10^8^–10^9^ CFU ml^−1^) after 4‐h incubation. From top to bottom: YFP fluorescence, mKate2 fluorescence, brightfield, overlay of images, zoom in of overlay images. From left to right: PS‐216 YFP, PS‐218 mKate2, PS‐218 YFP and PS‐218 mKate2 (isogenic – self interaction), PS‐216 YFP and PS‐218 mKate2 (non‐kin interaction). The white color in the overlay panel shows colocalization of YFP and mKate2. The scale is 25 μm for zoom‐in images and 100 μm for all others. (b) Visualization of responses in the context of biological pathways. For each comparison between *B. subtilis*‐inoculated and non‐inoculated plants (in columns for each strain or its combination, labeled as in (a)), gene sets enriched for up‐ or downregulated genes (*q*‐value < 0.1) are shown and designated as red triangles or blue triangles, respectively, after 2‐h incubation (10^8^–10^9^ CFU ml^−1^). MapMan ontology BINs were used to generate gene sets. See Supporting Information Table S8 for all results. (c) Examples of genes with intensified response after exposure to the non‐kin strains. Log_2_FC values are shown. Blue: downregulated genes, red: upregulated genes, according to the scale on the left. See Table S13 for gene IDs, annotations, and log_2_FC values.

To understand plant responses to colonizing bacterial communities, which is relevant for agricultural applications, we set up an experiment inoculating plants with two genetically different, previously categorized as non‐kin, and two isogenic (only differently fluorescently labeled) *B. subtilis* strains. When roots were inoculated with PS‐216 and PS‐218 strains separately, they both colonized the roots equally (Fig. 4a, first two columns). Similarly, when differentially labeled isogenic strains were combined, the resulting biofilm on the root contained similar abundances of each strain, with cells of each strain spatially well mixed (Fig. 4a). On the other hand, when non‐kin strains PS‐216 and PS‐218 were combined, we observed biofilm with segregation of the two strains (Fig. 4a). Moreover, one of the strains dominated on the roots, with only a small amount of biofilm formed by the other strain (Fig. 4a).

We next performed RNA‐seq analysis of roots at the time of uniform *B. subtilis* biofilm establishment (2 hpi, Fig. S5; Table S7A). In roots, the intensity of the transcriptional response to the non‐kin strain mixture was higher than that to the mixture of two isogenic, differentially labeled strains (Fig. 4b). Some receptor kinases (BINs 26.16 and 30.2.8.2) were specifically upregulated in the non‐kin interaction, as well as some enzymes from fatty acid biosynthesis (BIN 11.1.15) and heat shock proteins (BIN 20.2.1) (Fig. 4b; Table S8). In addition, 422 genes showed a more than 2‐fold stronger response in interactions with non‐kin strains than in monoculture or in the isogenic mixture (Table S7A). Among the more induced ones are two MYB transcription factors, salicylate carboxymethyltransferase, gibberellin 2‐oxidase 2, peroxidase, and beta‐galactosidase genes (Fig. 4c; Table S13).

### Systemic spread of *B. subtilis* to shoots is intensified in plants with silenced *StPti5*

**Fig. 5:**
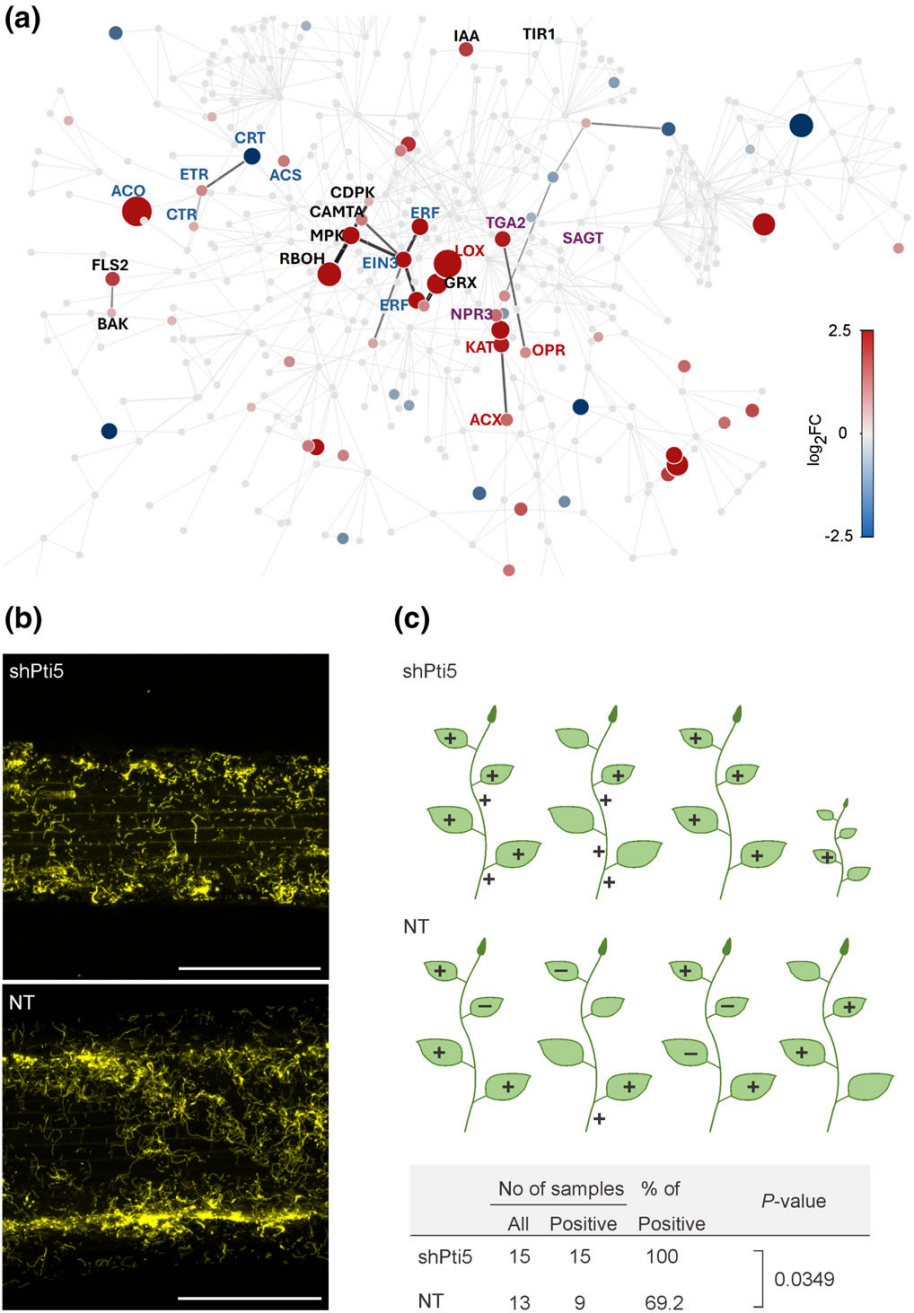
Ethylene response factor transcription factor StPti5 is required for balanced systemic colonization of potato plants. (a) Plant immune signaling modules that were regulated following incubation of roots with a non‐kin mixture of *Bacillus subtilis* strains. Genes and proteins are represented as nodes, and the interactions between them (protein–protein and protein–DNA interactions) are represented as edges of the network. Differentially expressed genes from ethylene (blue), JA (red), SA (violet), and other (black) signaling modules are shown. Black edge connects two differentially expressed genes. All non‐differentially expressed genes and edges are shown in gray. The size of the node indicates the difference in expression between PS‐216 and PS‐218 inoculated and non‐inoculated roots (log_2_FC). See Supporting Information Table S14 for the name and description of regulated genes, and Fig. S11 for information on central signaling modules that were regulated in potato roots in isogenic interaction (PS‐218 YFP + PS‐218 mKate2). (b) *Bacillus subtilis* PS‐218 YFP root colonization (yellow fluorescence) of transgenic plants with silenced *StPti5* (shPti5) and non‐transgenic potato plant (NT) after 2‐h incubation (10^8^–10^9^ CFU ml^−1^). Bars, 250 μm. Biofilm formation was also tested with two additional *B. subtilis* strains, PS‐216 YFP and PS‐68 YFP, and similar colonization patterns were observed for all strains (Fig. S13). (c) *B. subtilis* abundance in shPti5 L6 and NT potato plants. The scheme shows leaves of four shPti5 and four NT plants in which the presence of *B. subtilis* was (+) or was not (−) detected by qPCR in plants from soil, which were incubated for 2 h in PS‐218 YFP (10^8^–10^9^ CFU ml^−1^) before being planted and grown in soil for 1 wk. Leaves with no marks were not analyzed. The table presents a statistically significant difference in the percentage of positive samples (leaves, nodes, and internodes) between NT and shPti5 plants determined by Fisher's exact test. See Table S16 for details. The results were confirmed on another transgenic line, shPti5 L2 (Figs S12, S13; Table S17).

We next overlaid transcriptomic data with the PSS prior knowledge network, built from experimental data on protein–protein interactions, protein–DNA interactions, and metabolic pathways (skm.nib.si) (Bleker *et al*., 2024). The central signaling module regulated in the studied interaction was ethylene signaling, linked to regulated JA synthesis, SA signaling, and ROS signaling (Figs 5a, S11; Table S14). Thus, we focused on ERF transcription factors, as the role of ethylene signaling had not been addressed in depth in the context of plant interactions with beneficial microbes. Among ERFs, *StPti5* was found to be more than 50‐fold induced when plants were exposed to *B. subtilis* monocultures or two‐strain mixtures, already early after colonization (after 2‐h incubation, Tables S7B, S8). This could indicate its role as a regulatory hub in plant colonization and/or biofilm formation. To test this role, we produced a transgenic potato with reduced expression of *StPti5*. First, we followed biofilm formation in these transgenic plants with silenced *StPti5*. Results showed that StPti5 is not involved in biofilm formation on roots (Fig. 5b; Table S15), as no difference in biofilm formation was detected between non‐transgenic (NT) and *StPti5*‐silenced (shPti5) plants (Figs 5b, S12; Table S15).

*StPti5* was also upregulated in potato shoots after *B. subtilis* inoculation (Fig. 1b; Tables S7, S9). To test whether it plays a role in the long‐term maintenance of the *B. subtilis* community in the plant, we followed *B. subtilis* abundance in shoots of NT and shPti5 plants 1 wk after planting the inoculated plant in the soil. *Bacillus subtilis* abundance in shoots was higher in shPti5 plants, revealing that the balance in the abundance of the *B. subtilis* community was perturbed (Fig. 5c; Tables S16, S17).

### StPti5 also regulates the extent of potato root colonization by arbuscular mycorrhizal fungi

**Fig. 6:**
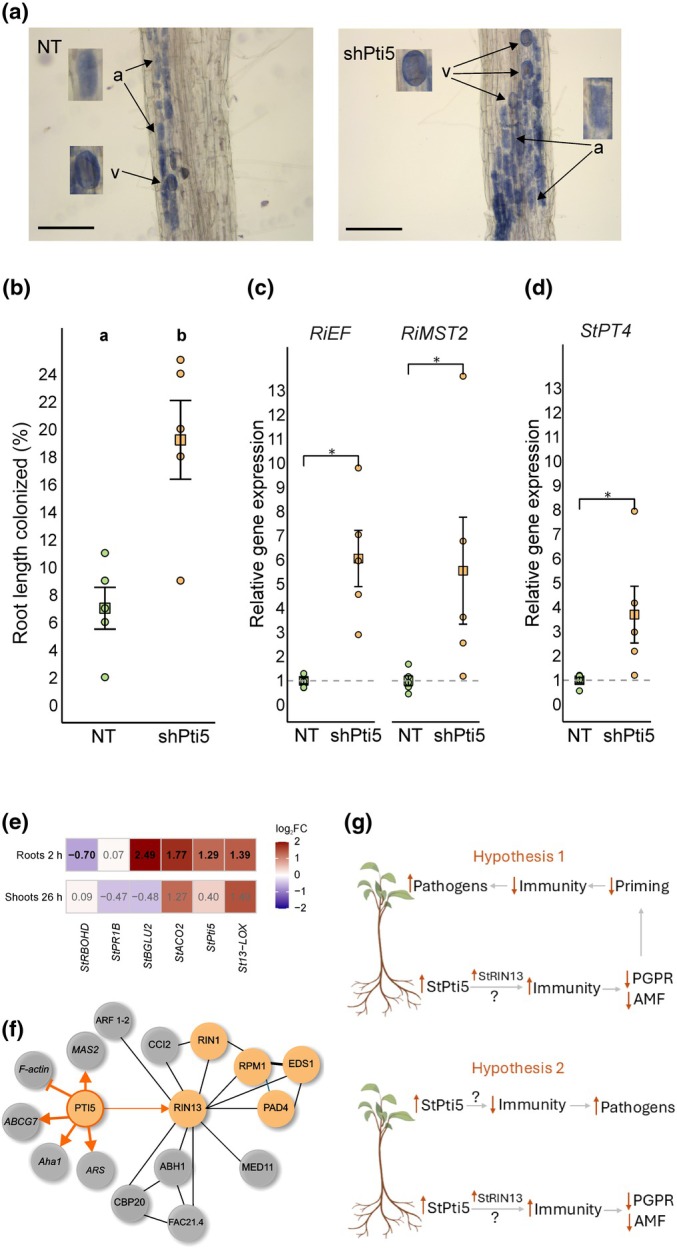
StPti5 regulates colonization of potato by diverse microorganisms through regulation of immune signaling. (a) Mycorrhizal colonization of potato roots in the non‐transgenic potato plant (NT) and the *StPti5*‐silenced genotype (shPti5). Representative images of mycorrhizal colonization by *Rhizophagus irregularis* in different potato genotypes 4 wk post‐inoculation. Fungal structures within the root cortex of potato plants are stained in blue (a, arbuscules; v, vesicles). Bars, 200 μm. (b) Percentage of root length colonized by the arbuscular mycorrhizal fungi *R. irregularis*. Percentage outcomes (*n* = 5) were analyzed using beta regression, with estimated marginal means (EMMs) computed for each genotype. Pair comparisons to the control group were conducted using Dunnett's method (dunnettx) in a one‐vs‐control design. Different letters indicate that the group means are statistically different from each other. (c) Expression analysis of *R. irregularis* constitutive gene (RiEF) and symbiosis marker genes, RiMST2 and (d) StPT4. Pairwise permutation test was used to determine the differences between the genotypes (*n* = 5). *P*‐values were adjusted using the Benjamini‐Hochberg (BH) procedure. For visualization purposes, relative gene expression was scaled to the average gene expression of NT. Individual measurements (circles), mean (squares), and SE are shown. Asterisks denote a statistically significant difference (*, *P* <0.05). The results for line L6 are shown and were confirmed in another transgenic line, L2 (Supporting Information Fig. S14; Table S17). (e) Response of the plant to flg22 does not resemble the response to *Bacillus subtilis* in potato. Gene expression response of *StRBOHD, StPR1B, StBGLU2, StACO2, StPti5*, and *St13‐LOX* genes in roots after 2 h and shoots after 26 h of flg22 treatment (Table S20). Pairwise permutation test was used to determine the differences between the treatment and control (*n* = 4). (f) StPti5 regulates transcription of St*RIN13* and, through that, immune signaling. Orange connection – StPti5 (PTI5) targets identified by DAP‐seq and RNA‐seq methods, black connections – connections of RIN13 as identified by STRING database search, orange nodes – proteins involved in immune signaling, gray nodes – all others. (g) A schematic illustration summarizing the hypotheses that could mechanistically explain the dual roles of StPti5—its function in regulating endophyte colonization and its role as a susceptibility factor. Elevated immune activation mediated by StPti5 in roots inoculated with beneficial bacteria may reduce the capacity of these bacteria to effectively colonize the root. Consequently, the systemic priming in the shoots may be diminished, leading to increased susceptibility to pathogens (hypothesis 1). Alternatively, the observed phenotypes may reflect distinct roles of StPti5 in roots and shoots, rather than a single unified mechanism (hypothesis 2).

Considering the role of StPti5 in regulating endophytic colonization by *B. subtilis*, we aimed to explore if this role was specific for this interaction or could be extended to other beneficial symbioses. We decided to explore the potential role of StPti5 in regulating the extension of root colonization by AMF. This symbiosis is known to be under plant control to keep the interaction under mutualistic levels and avoid excessive colonization (Ho‐Plágaro & García‐Garrido, 2022; Lidoy *et al*., 2023). With this aim, we explored the colonization levels and symbiosis functionality in NT and shPti5 lines. *Rhizophagus irregularis* successfully colonized potato roots, with intercellular hyphae, arbuscules, and vesicles present in the cortex (Fig. 6a). Remarkably, the extension of colonization, including the abundance of arbuscules and vesicles, was higher in the shPti5 lines (Fig. 6a). Indeed, the percentage of root length colonized by the fungus was more than twofold higher in the silenced lines, in agreement with the higher amount of fungal DNA in those plants (Fig. 6b; Table S18). To check, if the functionality of the mycorrhizal symbiosis was altered in those lines, we analyzed the expression levels of plant and fungal transporters involved in the nutrient exchange between the symbionts within the arbuscules: the fungal monosaccharide transporter RiMST2 (Fig. 6c; Table S19), and the potato phosphate transporter StPT4, both transcriptionally upregulated in arbusculated cells and thus regarded as markers for symbiotic functionality. Both markers were upregulated in the shPti5 lines (Figs 6c,d; Table S19), confirming the functionality of the symbiosis.

### *StRIN13* , a regulator of plant immune signaling, is a direct target of StPti5 transcription factor

We were next interested in the mechanism of StPti5 action. We first compared the *B. subtilis*‐triggered MTI response to the response triggered by flg22. *StBGLU* was strongly regulated, while *St13‐LOX* and *StPti5* were induced to a lesser extent (Fig. 6e, Table S20) 2 h post flg22 treatment if compared to the *B. subtilis*‐triggered response at the same time point (Fig. 1c; Tables S5, S6). Interestingly, no regulation of selected genes was detected in shoots 24 h post flg22 treatment, while shoots did respond to *B. subtili*s colonization at this time (Tables S2–S4). Thus, *B. subtilis* does not elicit the flg22‐mediated response associated with pathogen‐triggered MTI response.

To identify direct targets of StPti5, we performed DNA affinity purification sequencing (DAP‐seq). We in addition investigated the expression of downstream StPti5 target genes in a system in which the StPti5 protein is stabilized (Coll *et al*., 2024). For this, we selected the NahG‐Rywal‐PVY pathosystem and performed spatial RNA‐seq analysis on defined regions of the potato leaf (i.e. lesions) where the virus replicates and StPti5 accumulates substantially. The genes identified by both analyses were considered reliable targets of this transcription factor (Fig. 6f; Tables S21, S22). Functional analysis of identified target genes revealed an interesting link with immune signaling. Specifically, the RIN13, an RPM1‐interacting protein, was shown to promote PAD4 nuclear localization and, in this way, modulates immune signaling (Liu *et al*., 2020).

## Discussion

Microbial inoculants have been proven to be efficient and environmentally friendly alternatives to chemical pesticides and fertilizers (Alves de Andrade *et al*., 2023; Minchev *et al*., 2024). Current limitation in adopting such agricultural practices is their variable efficiency in the field due to a lack of understanding of the mechanisms involved in the regulation of plant colonization and maintenance of the plant microbiome (Tsotetsi *et al*., 2022; Lutz *et al*., 2023). Therefore, studying the mechanisms that regulate interactions between crop plants and microbes is crucial for ensuring the robustness of this plant protection approach. As the third most important crop in the world, exceptionally sensitive to a wide range of stresses, potato is one of the most suitable candidates for plant growth‐promoting rhizobacteria (PGPR)‐based products (Vilvert *et al*., 2022). We here established a model system for such studies, exploring selected *B. subtilis* soil isolates individually or in mixtures. Furthermore, we explored the potential extension of our findings by testing another widespread mutualistic interaction, the arbuscular mycorrhizal symbiosis.

*Bacillus subtilis* initially suppresses immune response in potato roots (e.g. by downregulation of diverse receptor kinases), thereby facilitating its own colonization, while it boosts induction of plant immunity‐related genes in shoots at a later stage (Fig. 2; Tables S7, S8). A similar response was also shown in other studies of plants with beneficials as part of IR phenomenon (Pieterse *et al*., 2014; Blake *et al*., 2021). Here, we show that live bacteria are required for triggering the plant response, as the conditioned media in which bacteria were growing did not trigger the response (Fig. S10). Moreover, the intensity of the plant response was dependent on the extent of biofilm formation on the potato root (Fig. 3). Both the mutant in surfactin production and the mutant in QS (also deficient in surfactin production) showed a reduced ability to form biofilm on roots (Fig. 3). Interestingly, the same strain was able to form floating biofilms (pellicles) (Špacapan *et al*., 2020), suggesting that the ComX QS control of the biofilm development also depends on plant factors. Besides residing on the plant roots, *B. subtilis* can also colonize the plant endophytically. Internalized bacteria have lower division rates and altered morphology (Figs 2, S7). The round bacterial form closely resembles L‐form bacteria, which are characterized by modified or absent cell walls (Allan *et al*., 2009; Leaver *et al*., 2009; Daulagala, 2021).

To get insights into the complexity of plant–microbe interactions, we extended our studies to the application of a mixture of two different *B. subtilis* strains to explore how bacterial diversity may influence the plant response. We studied pairs of strains with different kin discrimination types of intraspecies interactions–isogenic strains were predicted to be cooperative, and non‐kin strains to be antagonistic (Kraigher *et al*., 2022). We observed that non‐kin interaction of two *B. subtilis* strains enhances the plant response, particularly in immune and abiotic signaling components (Fig. 4; Tables S7, S8). These results imply that even minor taxonomic changes in the phytobiome can lead to substantial differences in the plant responses. Increasing evidence suggests that plants actively manage their microbiota to balance growth and immunity (Ku *et al*., 2024). It has been reported that *B. subtilis* can promote plant growth via direct pathways, such as producing phytohormones, for example, auxin, gibberellin, cytokinin, and abscisic acid (Karadeniz *et al*., 2006; Gamalero & Glick, 2011; Hamdache *et al*., 2011). Both strains used in this study produce auxins (Table S23). Interestingly, we here show that *B. subtilis* also regulates phytohormone metabolism *in planta*. Gibberellin 2‐oxidase 2 and cytokinin oxidase, which catalyze the irreversible degradation of cytokinin and gibberellin phytohormones, have been among the most induced genes by inoculation of non‐kin *B. subtilis* strains (Fig. 4c; Table S13). This could suggest that the equilibrium between growth and immunity is shifted toward immunity in plants inoculated by non‐kin *B. subtilis* strains, as a functional relation between lower activity of gibberellin signaling and reduced plant disease severity has previously been confirmed in potato (Križnik *et al*., 2017). In accordance, indole‐3‐acetic acid‐amido synthetase GH3.8 was induced in our study in *B. subtilis*‐inoculated plants at the later time point in both shoots and roots (Table S7B). GH3.8 prevents the accumulation of free IAA, which increases plant disease resistance, growth, and development (Xu *et al*., 2021).

Not much is known, however, about the mechanisms that enable beneficial microbes to successfully colonize plants while preventing colonization by harmful pathogens. MTI is known to be induced also when the plant is exposed to beneficials (Verbon *et al*., 2023). In Arabidopsis interactions with a beneficial *Pseudomonas* strain, suberin biosynthesis was downregulated to facilitate bacterial penetration (Verbon *et al*., 2023). Similarly, in our study, cell wall metabolism was downregulated in root colonization (Figs 2, 4; Table S8). In addition, synthesis of toxic terpenoids was downregulated in roots at the biofilm‐forming stage (Fig. 2). Suppression of toxic ROS is crucial for successful colonization of plants by microbes (Liu *et al*., 2024). We found ROS scavenger peroxidases (Table S7A) and ROS generator StRBOHD (Fig. 3) to be induced and repressed, respectively, following inoculation with *B. subtilis*. Several other studies also reported the increase of peroxidases in different plant–*Bacillus* interactions, and in some cases this induction has been associated with pathogen resistance (Lian *et al*., 2011; Chowdappa *et al*., 2013; Srivastava *et al*., 2016; Yasmin *et al*., 2016; Guo *et al*., 2019; Mehmood *et al*., 2021).

Plant colonization by beneficial microbes is frequently linked to the induction of SA, JA, and ethylene signaling pathways and transcriptional rewiring, of which MYB72, MYC2, and NPR1 transcription factors are the most studied ones and are proposed to be relevant for IR responses (Pieterse *et al*., 2014; Zamioudis *et al*., 2015). Here, we found upregulation of the St13‐LOX (Figs 1, 3) leading to JA synthesis, as previously described in other plant‐PGPR interactions (Cawoy *et al*., 2014; Zebelo *et al*., 2016; Wu *et al*., 2018). Similarly, several studies reported activation of the SA signaling pathway (Yu *et al*., 2022). We found NPR1 upregulated in shoots (Table S7B). In corroboration with previous studies, we also identified several MYB transcription factors induced in both roots and shoots (Table S7B).

Modulation of ethylene signaling in plant microbiome interactions has been pinpointed as a central element in their regulation and their impact on plant responses to stress (Ravanbakhsh *et al*., 2018). For example, ethylene has been proposed to regulate biofilm formation and root colonization by beneficial bacteria (Berrabah *et al*., 2018; Carlew *et al*., 2025) and the arbuscular mycorrhizal symbiosis (Das *et al*., 2025), and it is required for IR by beneficial bacteria and fungi (Knoester *et al*., 1999; Lidoy *et al*., 2025). We also detected induction of the ethylene signaling pathway in *B. subtilis*‐inoculated roots, including ETR, EIN3, and several ERF transcription factors (Figs 5, S11; Table S7B). Moreover, 1‐aminocyclopropane‐1‐carboxylate oxidase, which catalyzes the final step in ethylene biosynthesis, was induced in all tissues and time points in our study, further suggesting involvement of the ethylene signaling pathway in potato response to *B. subtilis* (Figs 5, S11; Table S7B).

ERF transcription factors, members of the AP2/ERF family active under ethylene signaling, play prominent roles in plant immunity, although the functions of most remain unknown (Pieterse *et al*., 2014; Chowdhury *et al*., 2015; Huang *et al*., 2016; Wu *et al*., 2018). A different set of ERFs was regulated in roots and shoots in our study. Among them, ERF transcription factor *StPti5* was strongly upregulated in all studied conditions. StPti5 was first identified in tomato as an interacting partner of the *R* gene *Pto* that confers resistance against the bacterial pathogen *Pseudomonas syringae* pv tomato (Zhou *et al*., 1997; He *et al*., 2001). On the other hand, we have previously shown that StPti5 negatively regulates potato defense response to pathogens PVY and *Ralstonia solanacearum*, suggesting its role as a susceptibility factor in potato immunity (Coll *et al*., 2024). Interestingly, *StPti5* is transcriptionally regulated by StEIN3 in an SA‐dependent manner and is regulated by autophagy degradation at the protein level (Coll *et al*., 2024). Results of this study suggest that StPti5 has a negative regulatory role also in the maintenance of endophytic community in potato, as both *B. subtilis* abundance in systemic tissues and mycorrhizal colonization of roots were increased in *StPti5*‐silenced potato plants (Figs 5, 6). We here additionally show that, among others, *StRIN13* is a direct target of StPti5 (Fig. 6f, Tables S21, S22). In Arabidopsis, RIN13 was shown to promote nuclear accumulation of PAD4, thereby modulating the immune response (Liu *et al*., 2020), which may be one mechanism explaining the observed results. There are, however, other possible explanations for Pti5 function, for example, altered root permeability or changes in exudate composition mediated by other targets of StPti5. In addition to StRIN13, we have identified other direct targets of StPti5 (Fig. 6f; Tables S21, S22), which might contribute to the regulation of microbial colonization in our interaction. Work in legumes suggested that the F‐actin coordinates numerous cellular processes in the development of nitrogen‐fixing nodules (Zhang *et al*., 2019). ABCG7, an ABC transporter, affects the secretion of metabolites and signaling molecules into the rhizosphere, thereby influencing microbial recruitment (Badri *et al*., 2009). AHA1, a plasma membrane H^+^‐ATPase, regulates rhizosphere acidification and root physiological processes (Fan *et al*., 2024) that can shape the local microbial environment and potentially impact microbial colonization. Increased immune activation driven by StPti5 during root interactions with beneficial microbes reduces their ability to colonize the root. As a consequence, systemic priming in shoots is reduced, which in turn leads to increased susceptibility to pathogens–the function previously attributed to StPti5. However, the observed phenotypes could also reflect distinct roles of StPti5 in roots and shoots, rather than a single unified mechanism (Fig. 6g).

In natural environments, plant roots interact with highly diverse and dynamic microbial communities, and the outcomes of such interactions can vary depending both on plant genotype and on microbiome composition. To better reflect conditions found in natural environments, we used three different *B. subtilis* strains, their communities and two unrelated potato genotypes. Future studies incorporating even more diverse plant genetic backgrounds and community‐level microbial interactions will be important for assessing the generality and ecological relevance of the mechanisms described here.

To summarize, high StPti5 levels both increase potato susceptibility to pathogens and reduce its capability to interact with beneficial organisms. *StPti5* thus represents a target for future breeding efforts leading toward sustainable potato production. Our findings establish a cornerstone for understanding how beneficial microbes and plants communicate to regulate endophytic colonization and maintain mutualism, and identify a means to improve colonization by beneficial microbes through silencing of *StPti5*.

## Competing interests

None declared.

## Author contributions

TL, MJP, IM‐M and KG contributed to the conceptualization and design of the study. TL, BK, KP, KS, TGK, MZ, MP, PS, AV, VL, TMP, JMG, EA, JMF‐Z, MJP, IM‐M and KG contributed to the acquisition, analysis, or interpretation of data. TL and KG contributed to the drafting of the manuscript. TL, BK, KP, KS, TGK, AV, VL, TMP, EA, JMF‐Z, JMG, MK, MZ, MP, PS, MJP, IM‐M, ŠB and KG involved in the critical revision of the manuscript for important intellectual content. IM‐M and KG contributed equally to this work and shared last authorship.

## Disclaimer

The New Phytologist Foundation remains neutral with regard to jurisdictional claims in maps and in any institutional affiliations.

## Supporting information


**Fig. S1** Potato inoculation with *Bacillus subtilis*.
**Fig. S2** Biofilm on potato cv Rywal roots imaged at different time points after incubation in *Bacillus subtilis* culture.
**Fig. S3** Biofilm on potato cv Désirée roots after inoculation with two *Bacillus subtilis* strains.
**Fig. S4** Potato cv Rywal response to *Bacillus subtilis*.
**Fig. S5** Number of genes, regulated by *Bacillus subtilis* in plants incubated in *B. subtilis* culture (10^8^–10^9^ CFU ml^−1^).
**Fig. S6** *Bacillus subtilis* internalization within plant roots.
**Fig. S7** Observation of *Bacillus subtilis* cells in potato stems and leaves after internalization.
**Fig. S8** Morphology of *Bacillus subtilis* cells in liquid culture and potato leaf.
**Fig. S9** Biofilm formation on potato roots inoculated with *Bacillus subtilis* mutants that have atenuated surfactin production.
**Fig. S10** *Bacillus subtilis*‐produced secondary metabolites in the medium do not induce the same response in potato roots as inoculation with live bacteria.
**Fig. S11** Regulated central signaling modules in potato roots in isogenic and non‐kin interactions.
**Fig. S12** *Bacillus subtilis* root colonization of transgenic potato cv Rywal lines with silenced *StPti5* (shPti5 L2 and L6) and non‐transgenic (NT) potato plant.
**Fig. S13** Biofilm formation on transgenic potato lines with silenced StPti5 (shPti5 L2 and shPti5 L6) and non‐transgenic (NT) potato roots inoculated with two different *Bacillus subtilis* strains.
**Fig. S14** Mycorrhizal colonization of potato roots in non‐transgenic potato plant (NT) and Pti5‐silenced genotype line 2 (shPti5).
**Methods S1** Plant growth conditions.
**Methods S2** *Bacillus subtilis* strains.
**Methods S3** *Bacillus subtilis* IAA production detection.
**Methods S4** Plant inoculations.
**Methods S5** Mycorrhizal symbiosis establishment and quantification.
**Methods S6** Confocal microscopy settings.
**Methods S7** Transmission electron microscopy sample preparation.
**Methods S8** Nucleic acid isolation and purification.
**Methods S9** Statistical analysis of qPCR, microbial abundance and imaging data.
**Methods S10** RNA‐Seq analysis.
**Methods S11** DAP‐seq.

**Table S1** Selected genes and corresponding primers and probe sequences for expression analyses with quantitative PCR.
**Table S2** Standardized qPCR gene expression data.
**Table S3** Standardized qPCR gene expression data for additional *Bacillus subtilis* strain PS‐68.
**Table S4** Standardized qPCR gene expression data for additional genotype Désirée.
**Table S5** Standardized qPCR gene expression data of Experiment 1.
**Table S6** Standardized qPCR gene expression data of Experiment 2.
**Table S7** Differential gene expression table for comparisons between plants inoculated with *Bacillus subtilis* and non‐inoculated plants, and for selected genes regulated in roots at the time of biofilm formation and shoots after established root colonization.
**Table S8** Results of gene set enrichment analysis.
**Table S9** Technical confirmation of RNA‐seq by qPCR.
**Table S10** The amount of biofilm formation of *Bacillus subtilis* mutants on potato roots.
**Table S11** Relative gene expression in potato inoculated with *Bacillus subtilis* mutants that have attenuated surfactin production.
**Table S12** Expression of selected potato genes induced by conditioned medium in which *Bacillus subtilis* was growing.
**Table S13** Selected genes with intensified response to the non‐kin strains.
**Table S14** Plant stress signaling (PSS) prior knowledge network node information with corresponding RNA‐seq differential expression.
**Table S15** The amount of biofilm formation on potato roots for non‐transgenic (NT) and shPti5 plants.
**Table S16** *Bacillus subtilis* abundance in shPti5 L6 and non‐transgenic (NT) potato plants determined by qPCR.
**Table S17** *Bacillus subtilis* abundance in shPti5 L2 and non‐transgenic (NT) potato plants determined by qPCR.
**Table S18** Arbuscular mycorrhizal fungi colonization in StPti5‐silenced lines.
**Table S19** Molecular assessment of mycorrhizal colonization.
**Table S20** Expression of selected potato genes after flagellin treatment.
**Table S21** DAP‐seq results of StPti5 binding to potato cv Désirée genome library and mapped to PGSC potato gene model (v.4.03), revealing all available binding sites within whole potato genome.
**Table S22** Differentially expressed genes in StPti5‐silenced NahG vs NahG potato plants after PVY infection.
**Table S23** The production of indole acetic acid (IAA) was assessed following the protocol of Pramanik *et al*. (2023).
Please note: Wiley is not responsible for the content or functionality of any Supporting Information supplied by the authors. Any queries (other than missing material) should be directed to the *New Phytologist* Central Office.

## Data Availability

The RNA‐seq and DAP‐seq raw Illumina reads have been deposited in NCBI's Gene Expression Omnibus under accession nos. GSE232028 and GSE313812. All data analysis scripts, input and processed data files for qPCR, RNA‐seq (including the used *S. tuberosum* genome assembly, genome annotation, and GSEA gene set files) and network analyses are publicly available in the GitHub repository at https://github.com/NIB‐SI/Bacillus‐Pti5/ (v.1.0.0 archived via Zenodo, doi: 10.5281/zenodo.17728235). The transgenic bacterial strains created for this study are available from the corresponding author upon reasonable request. Microscopy datasets are available as TIFF and LIF files on Zenodo (doi: 10.5281/zenodo.18174134) and can be opened with las x software (https://www.leica‐microsystems.com/products/microscope‐software/p/leica‐las‐x‐ls/downloads/).
