# Supplementary material for: ERF transcription factor StPti5 is a regulator of endophyte community maintenance in potato
Source: New Phytol. 2026 May 24;251(3):1344–60. doi: 10.1111/nph.71240 (PMC13326511; doi:10.1111/nph.71240)
Supplement: Supplementary file 1 — Fig. S1 Potato inoculation with Bacillus subtilis. Fig. S2 Biofilm on potato cv Rywal roots imaged at different time points after incubation in Bacillus subtilis culture. Fig. S3 Biofilm on potato cv Désirée roots after inoculation with two Bacillus subtilis strains. Fig. S4 Potato cv Rywal response to Bacillus subtilis. Fig. S5 Number of genes, regulated by Bacillus subtilis in plants incubated in B. subtilis culture (108–109 CFU ml−1). Fig. S6 Bacillus subtilis internalization within plant roots. Fig. S7 Observation of Bacillus subtilis cells in potato stems and leaves after internalization. Fig. S8 Morphology of Bacillus subtilis cells in liquid culture and potato leaf. Fig. S9 Biofilm formation on potato roots inoculated with Bacillus subtilis mutants that have atenuated surfactin production. Fig. S10 Bacillus subtilis‐produced secondary metabolites in the medium do not induce the same response in potato roots as inoculation with live bacteria. Fig. S11 Regulated central signaling modules in potato roots in isogenic and non‐kin interactions. Fig. S12 Bacillus subtilis root colonization of transgenic potato cv Rywal lines with silenced StPti5 (shPti5 L2 and L6) and non‐transgenic (NT) potato plant. Fig. S13 Biofilm formation on transgenic potato lines with silenced StPti5 (shPti5 L2 and shPti5 L6) and non‐transgenic (NT) potato roots inoculated with two different Bacillus subtilis strains. Fig. S14 Mycorrhizal colonization of potato roots in non‐transgenic potato plant (NT) and Pti5‐silenced genotype line 2 (shPti5). Methods S1 Plant growth conditions. Methods S2 Bacillus subtilis strains. Methods S3 Bacillus subtilis IAA production detection. Methods S4 Plant inoculations. Methods S5 Mycorrhizal symbiosis establishment and quantification. Methods S6 Confocal microscopy settings. Methods S7 Transmission electron microscopy sample preparation. Methods S8 Nucleic acid isolation and purification. Methods S9 Statistical analysis of qPCR, microbial abundance and imagi [file NPH-251-1344-s002.docx]

**Supporting Information**

**ERF transcription factor StPti5 is a regulator of endophyte community maintenance in potato**

**Tjaša Lukan^1*^, Karmen Pogačar^1,2^, Barbara Kraigher^3^, Katja Stare^1^, Teja Grubar Kovačič^1^, Maja Zagorščak^1^, Marko Petek^1^, Polonca Stefanic^3^, Anže Vozelj^1,2^, Valentina Levak^1,2^, Tjaša Mahkovec Povalej^1^,** **Juan M. García^4^, Maria J. Pozo^4^, Eva Álvarez^5^, José M. Franco-Zorrilla^5^, Maja Križnik^1^, Špela Baebler^1^, Ines Mandić-Mulec^3+^, Kristina Gruden^1+*^**

^1^Department of Biotechnology and Systems Biology, National Institute of Biology, 1000 Ljubljana, Slovenia

^2^Jožef Stefan International Postgraduate School, Jamova cesta 39, 1000 Ljubljana, Slovenia

^3^Chair of Microbial Ecology and Physiology, Department of Microbiology, Biotechnical Faculty, University of Ljubljana, 1000 Ljubljana, Slovenia

^4^Department of Soil and Plant Microbiology, Estación Experimental del Zaidín (CSIC), Granada, Spain

^5^Department of Plant Molecular Genetics, Centro Nacional de Biotecnología-CSIC, Darwin 3, 28049-Madrid, Spain

**^+-^**These authors share last authorship

*-corresponding authors

Tjaša Lukan ([tjasa.lukan@nib.si](mailto:tjasa.lukan@nib.si)), Kristina Gruden ([kristina.gruden@nib.si](mailto:kristina.gruden@nib.si))

Article acceptance date: 24 March 2026


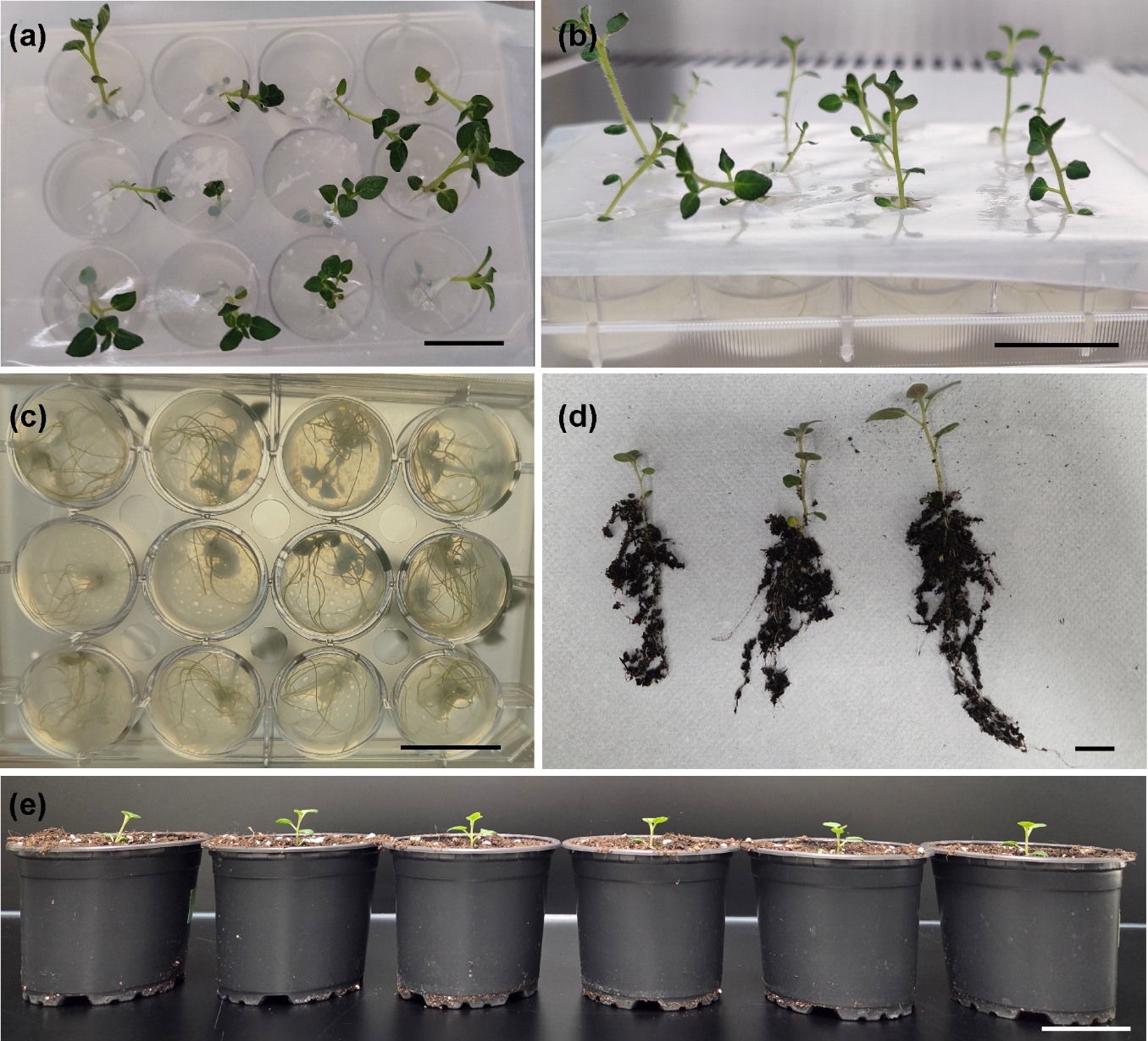


**Fig. S1: Potato inoculation with *B. subtilis.*** (a) – (c) Roots of two weeks old potato plants from tissue cultures were incubated in *B. subtilis* culture for a specified time at 11 rpm (see Plant inoculations methods section for more details). Scale is 2 cm. (d) – (e) For the experiments in the soil, plants were planted in soil after 2 h incubation in bacteria and sampled after one week. Scales are 1.5 cm (d) and 4 cm (e).


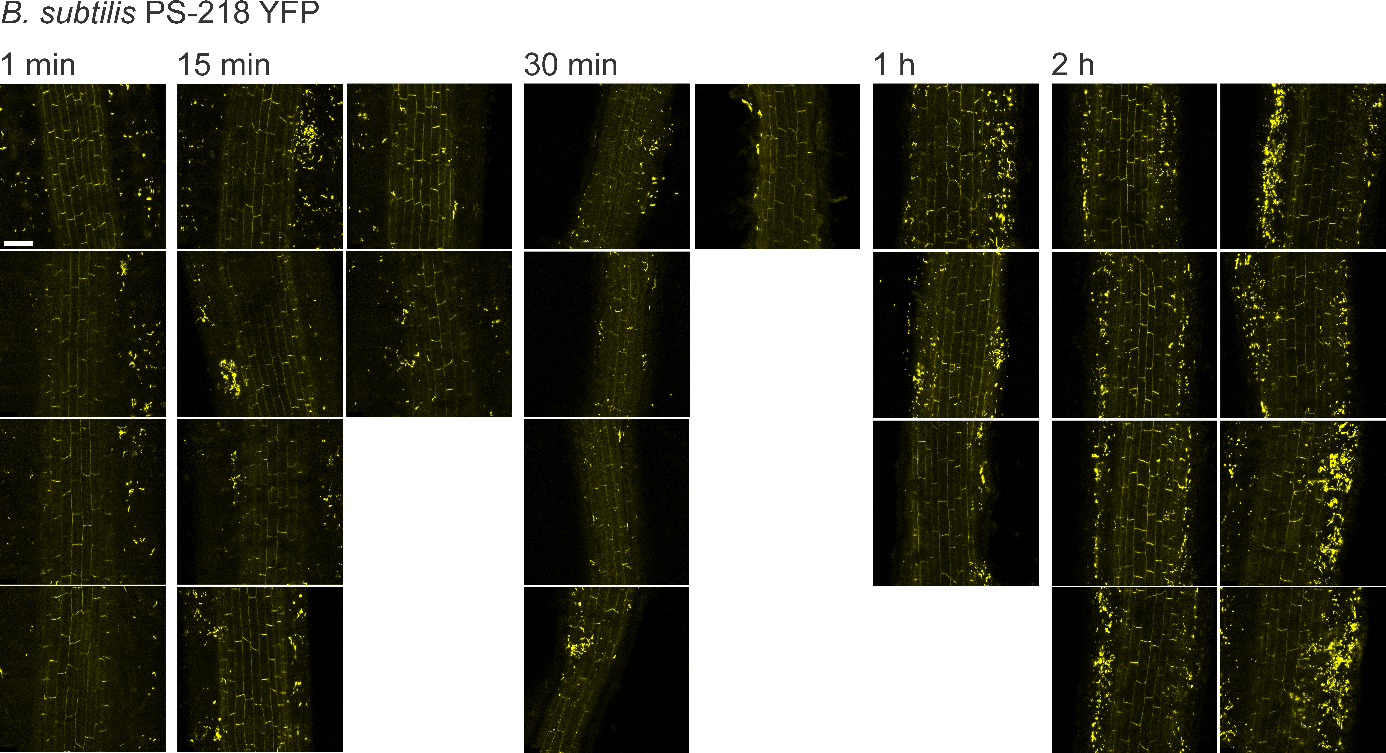


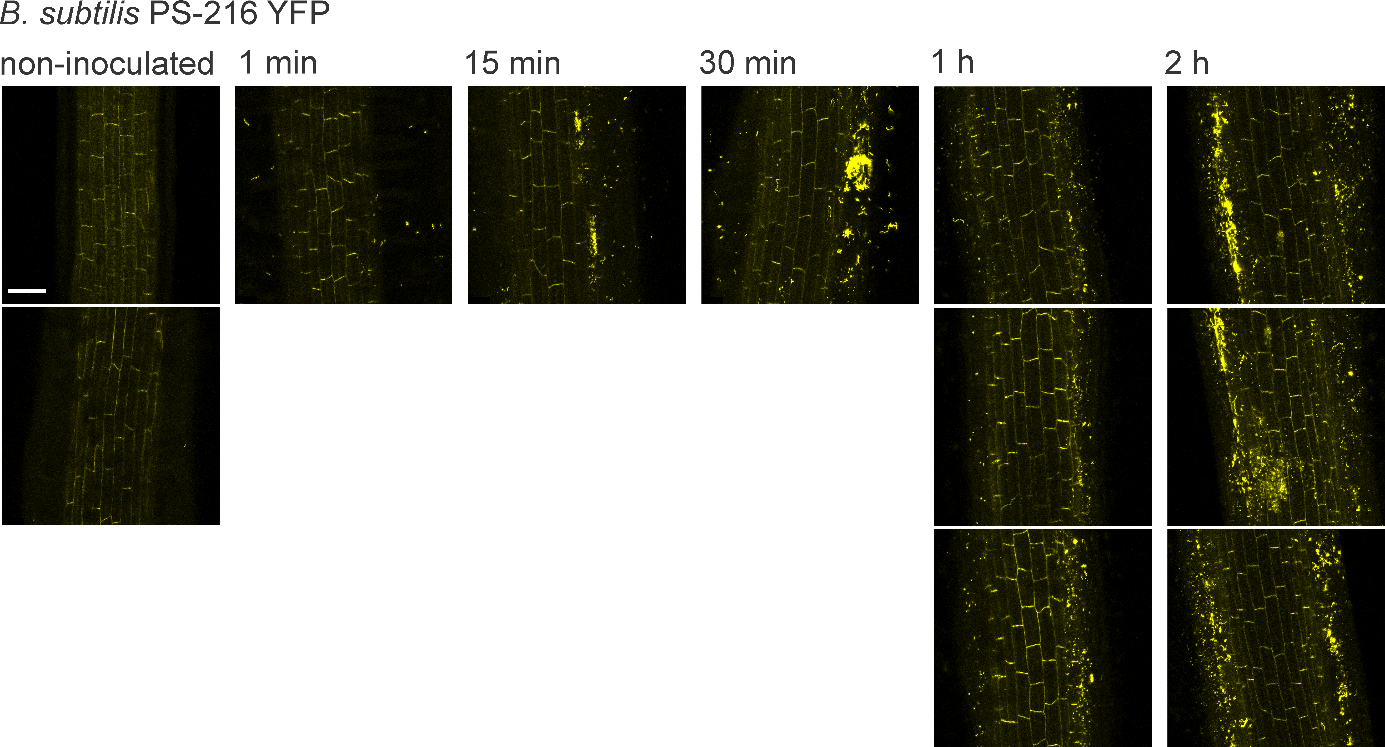


**Fig. S2: Biofilm on potato cv. Rywal roots imaged at different time points after incubation in *B. subtilis* culture.** Biofilm formation on potato cv. Rywal roots was imaged by following YFP fluorescence of YFP-tagged *B. subtilis* PS-218 or PS-216 culture after 1 min, 15 min, 30 min, 1 h and 2 h incubation in bacteria culture (10^8^ to 10^9^ CFU/mL). YFP fluorescence is colored as yellow. Note that cell walls are visible in YFP channel due to autofluorescence. Scale is 100 µm. Additional images are available on Zenodo (<https://doi.org/10.5281/zenodo.18174134>).


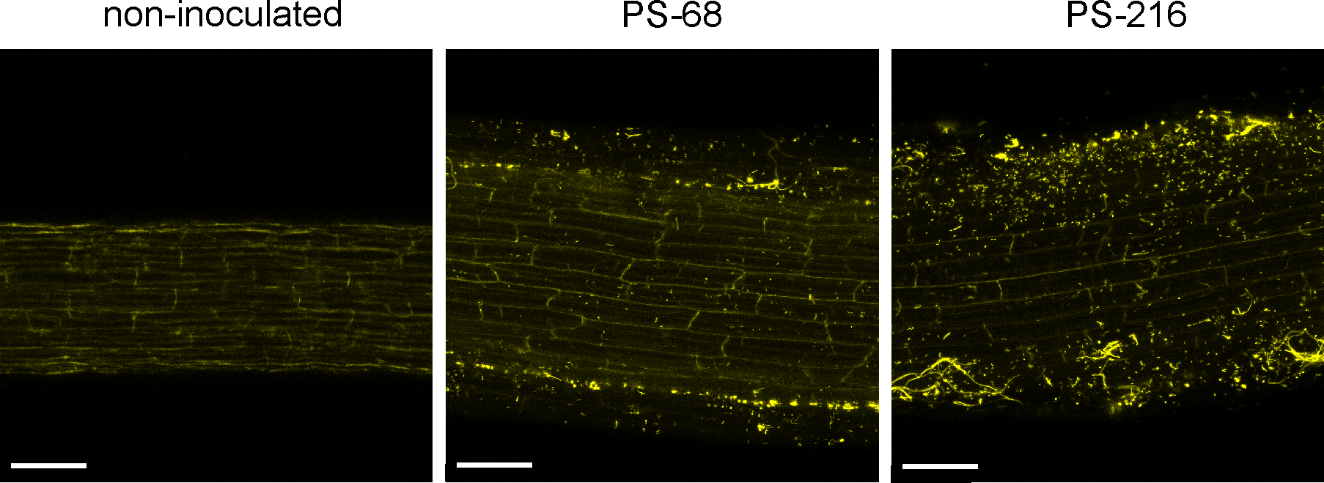


**Fig. S3: Biofilm on potato cv. Désirée roots after inoculation with two *B. subtilis* strains.** Biofilm formation on potato cv. Désirée roots was imaged by following YFP fluorescence of YFP-tagged *B. subtilis* PS-68 or PS-216 cultures after 2-h incubation in bacteria culture (10^8^ to 10^9^ CFU/mL). YFP fluorescence is colored as yellow. Note that cell walls are visible in YFP channel due to autofluorescence. Scale is 100 µm. Additional images are available on Zenodo (<https://doi.org/10.5281/zenodo.18174134>).


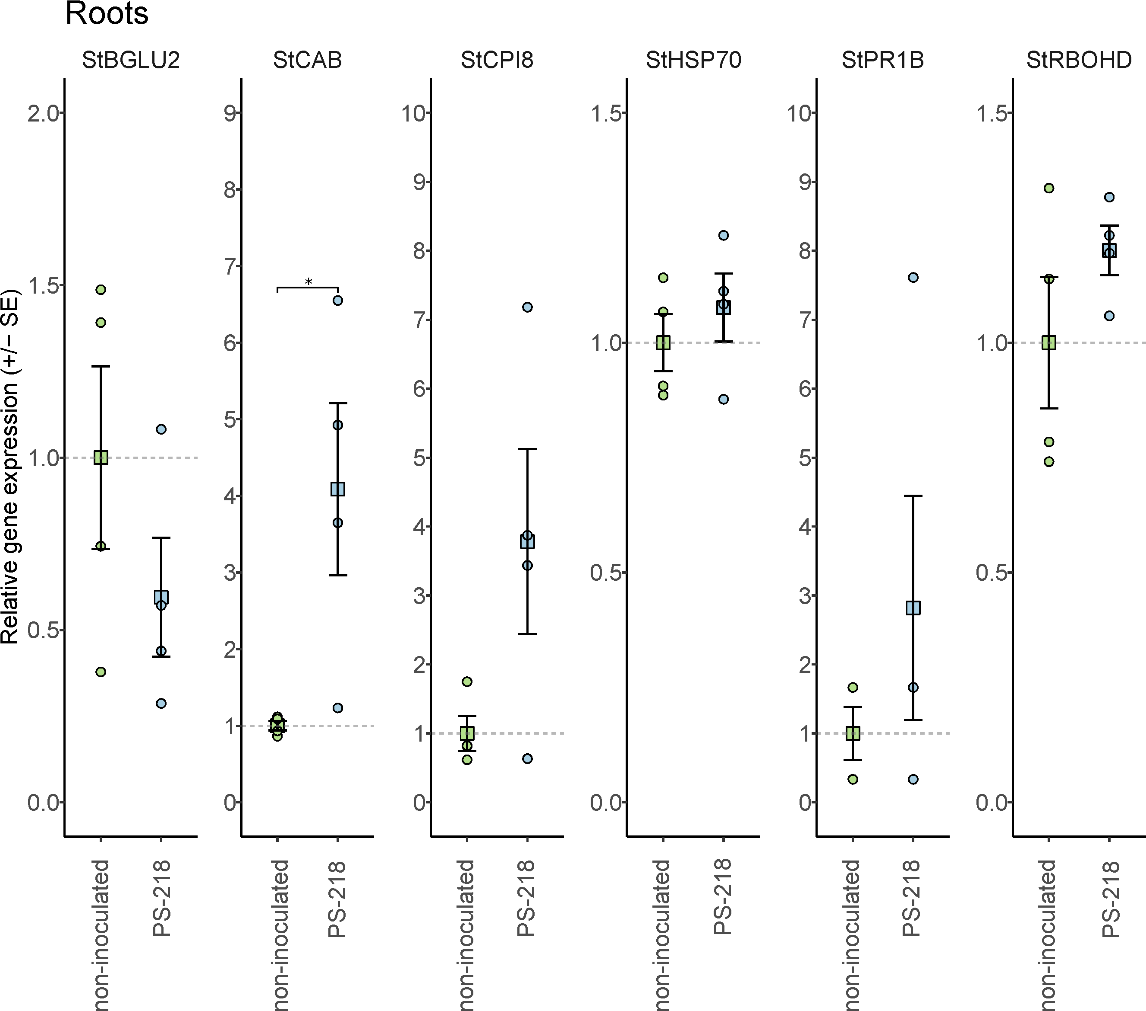


**(b)**

**(a)**


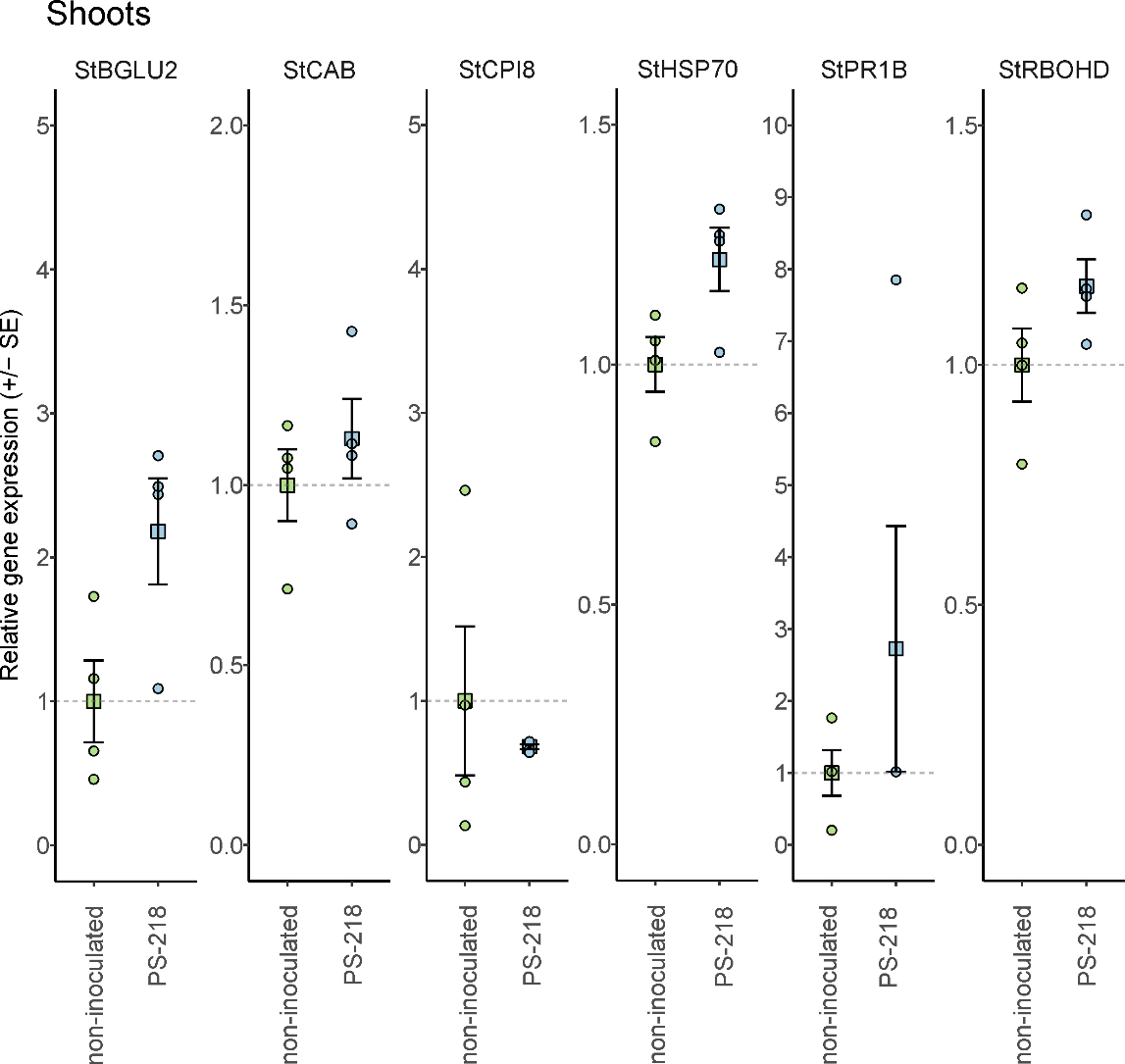

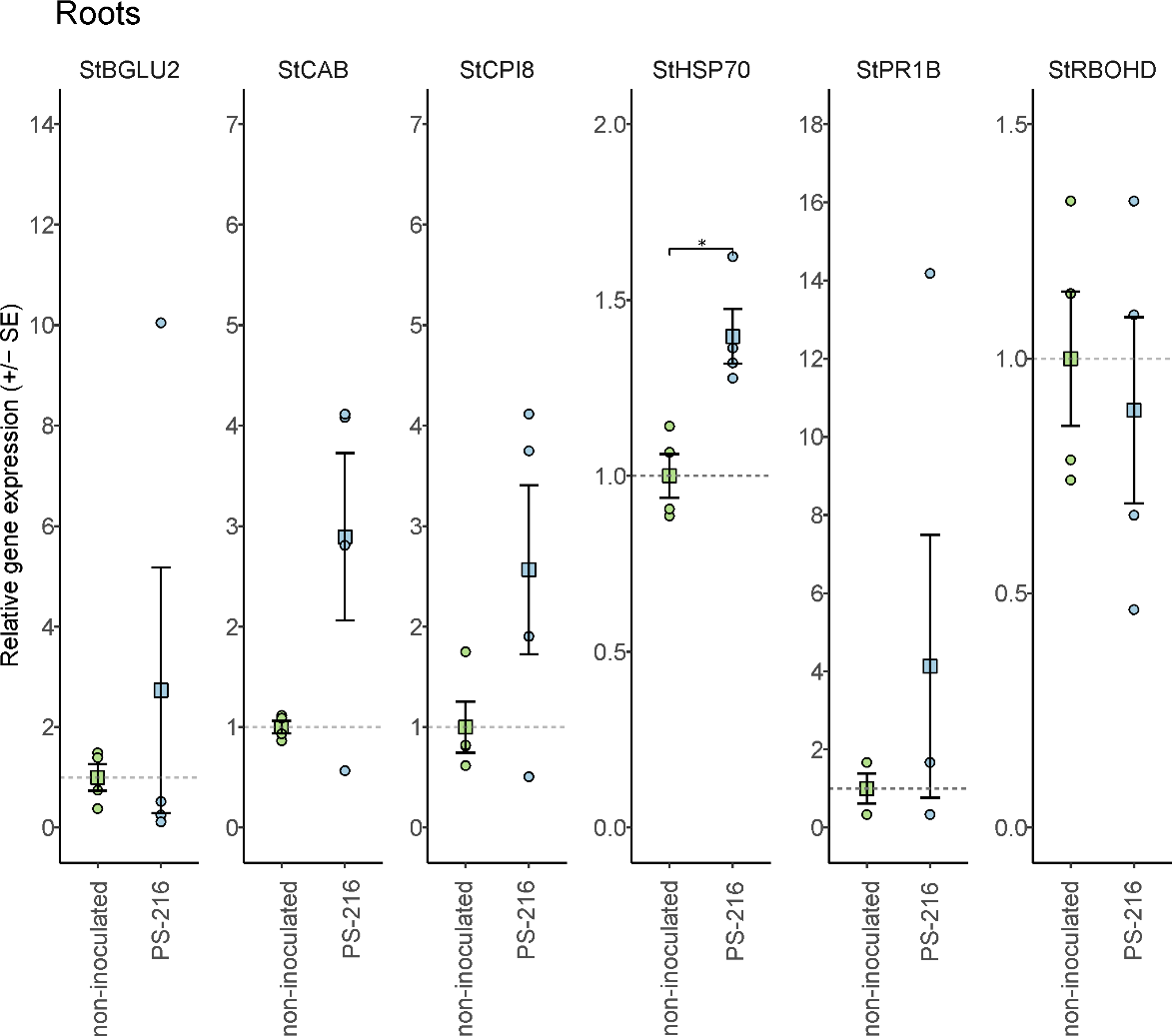


**(d)**

**(c)**


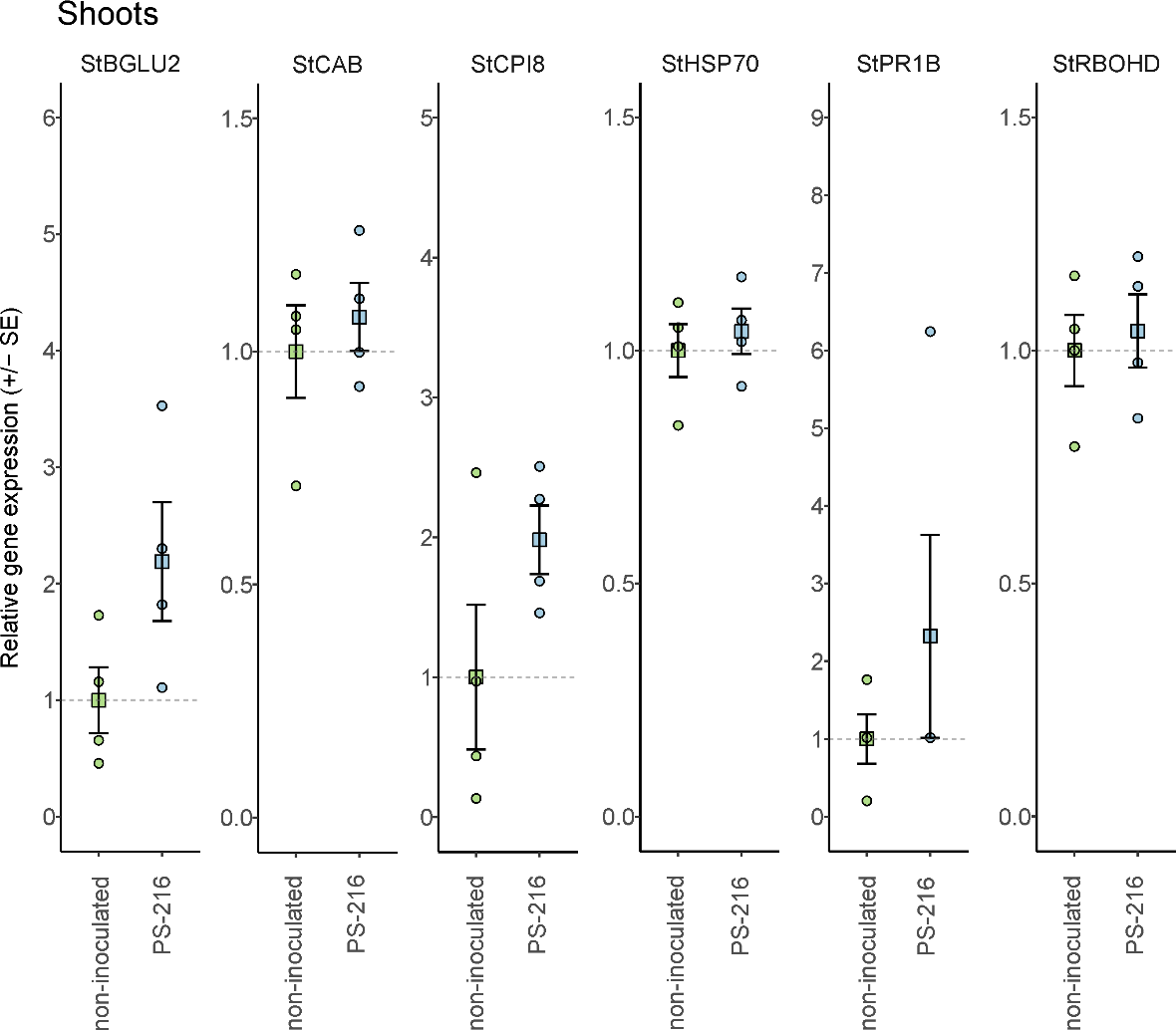


**Fig. S4: Potato cv. Rywal response to *B. subtilis.*** Relative expression values for six selected potato genes *StBGLU2*, *StCAB*, *StCPI8*, *StHSP70*, *StPR1B* and *StRBOHD* in (a) roots of *B. subtilis* PS-218 YFP, (b) shoots of *B. subtilis* PS-218 YFP, (c) roots of *B. subtilis* PS-216 YFP and (d) shoots of *B. subtilis* PS-216 YFP and non-inoculated plants, after overnight incubation in bacterial culture (10^7^ CFU/ml). Permutational t-test was used to determine differences between treatments (n = 4). P-values were adjusted using the Benjamini-Hochberg (BH) procedure. For visualization purpose, relative gene expression was scaled to the average gene expression of non-inoculated group. Individual measurements (circles), mean (squares) and standard error are shown. Asterisks (*) denote a statistically significant difference (p-value < 0.05). Experimental data are available in Table S2.


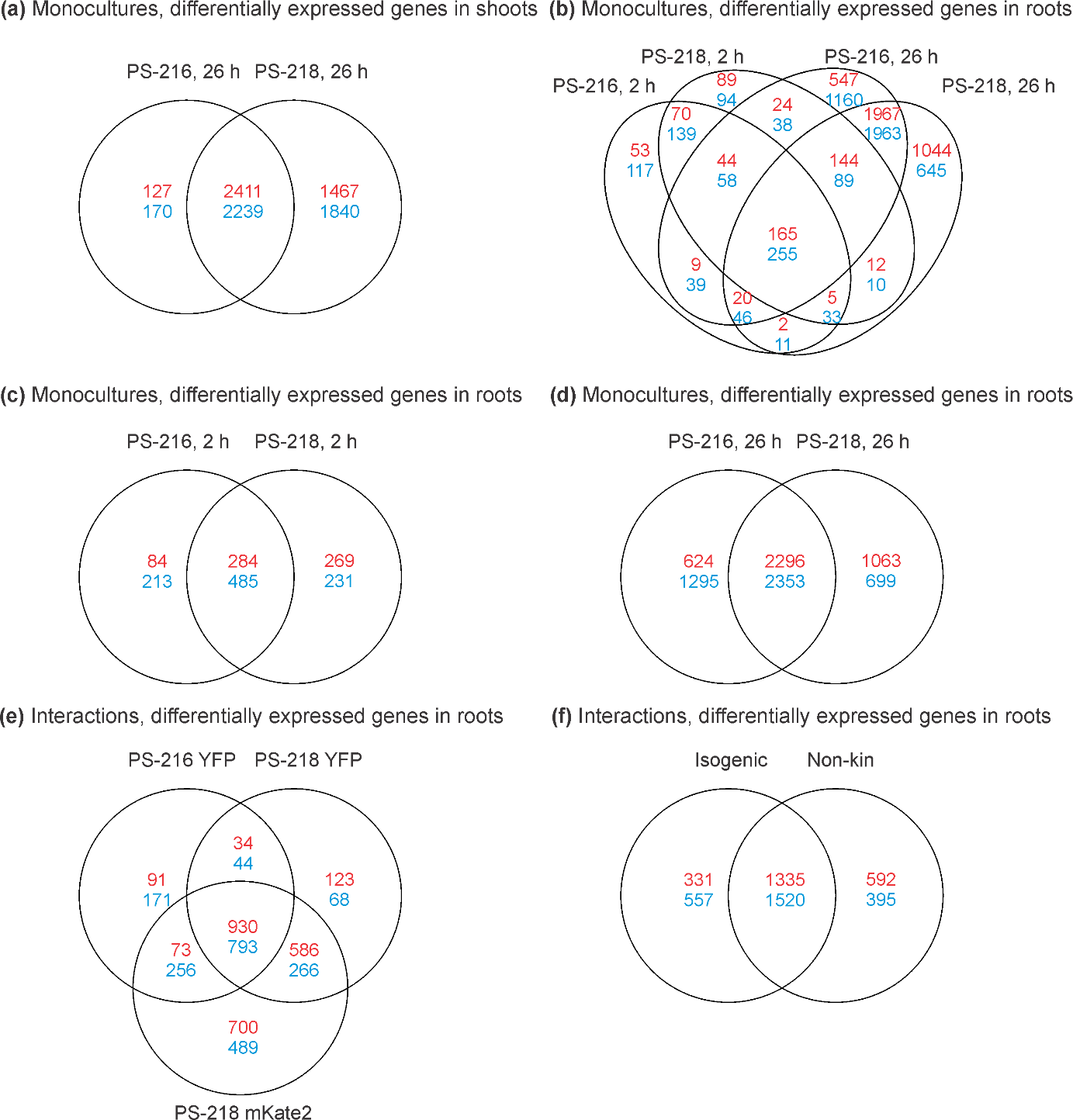


**Fig. S5: Number of genes, regulated by *B. subtilis* in plants incubated in *B. subtilis* culture (10^8^ - 10^9^ CFU/mL).** (a) Shoots of monocultures experiment comparing strains PS-216 and PS-218 after 26-h incubation. (b) Roots of monocultures experiment comparing strains PS-216 and PS-218 after 2-h and 26-h incubation. (c) Roots of monocultures experiment comparing strains PS-216 and PS-218 after 2-h incubation. (d) Roots of monocultures experiment comparing strains PS-216 and PS-218 after 26-h incubation. (e) Roots of interactions experiment comparing strains PS-216 YFP, PS-218 YFP and PS-218 mKate2 after 2-h incubation. (f) Roots of interactions experiment comparing strains in isogenic and non-kin interactions after 2-h incubation. Numbers of unique and common differentially expressed genes are shown with absolute log_2_ fold change above 1 and FDR-adjusted p-values below 0.05 are counted and presented in blue (downregulated) or red (upregulated). PS-216: PS-216 YFP, PS-218: PS-218 mKate2, isogenic: PS-218 YFP + PS-218 mKate2, non-kin: PS-216 YFP + PS-218 mKate2. See Table S7A for results on individual genes.


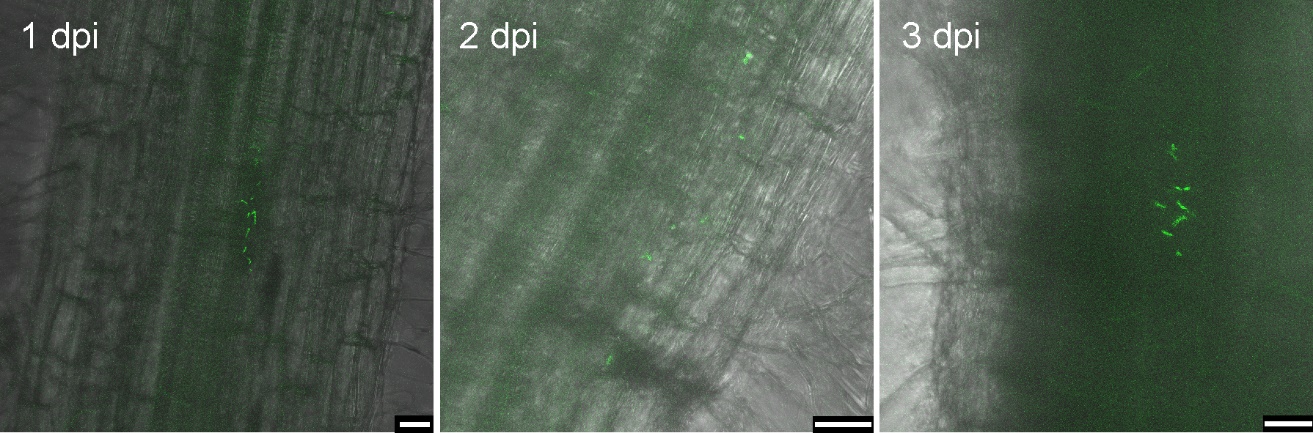


**Fig. S6: *B. subtilis* internalization within** **plant roots.** Potato cv. Rywal tissue cultures were inoculated with *B. subtilis* PS-216 YFP (see methods section Plant Inoculations). Surface-sterilized roots were observed using confocal microscopy, and bacterial cells were visualized by following YFP fluorescence for up to three days of root–bacterium contact. Scale is 25 µm. Microscopy lif files are available on Zenodo (<https://doi.org/10.5281/zenodo.18174134>).


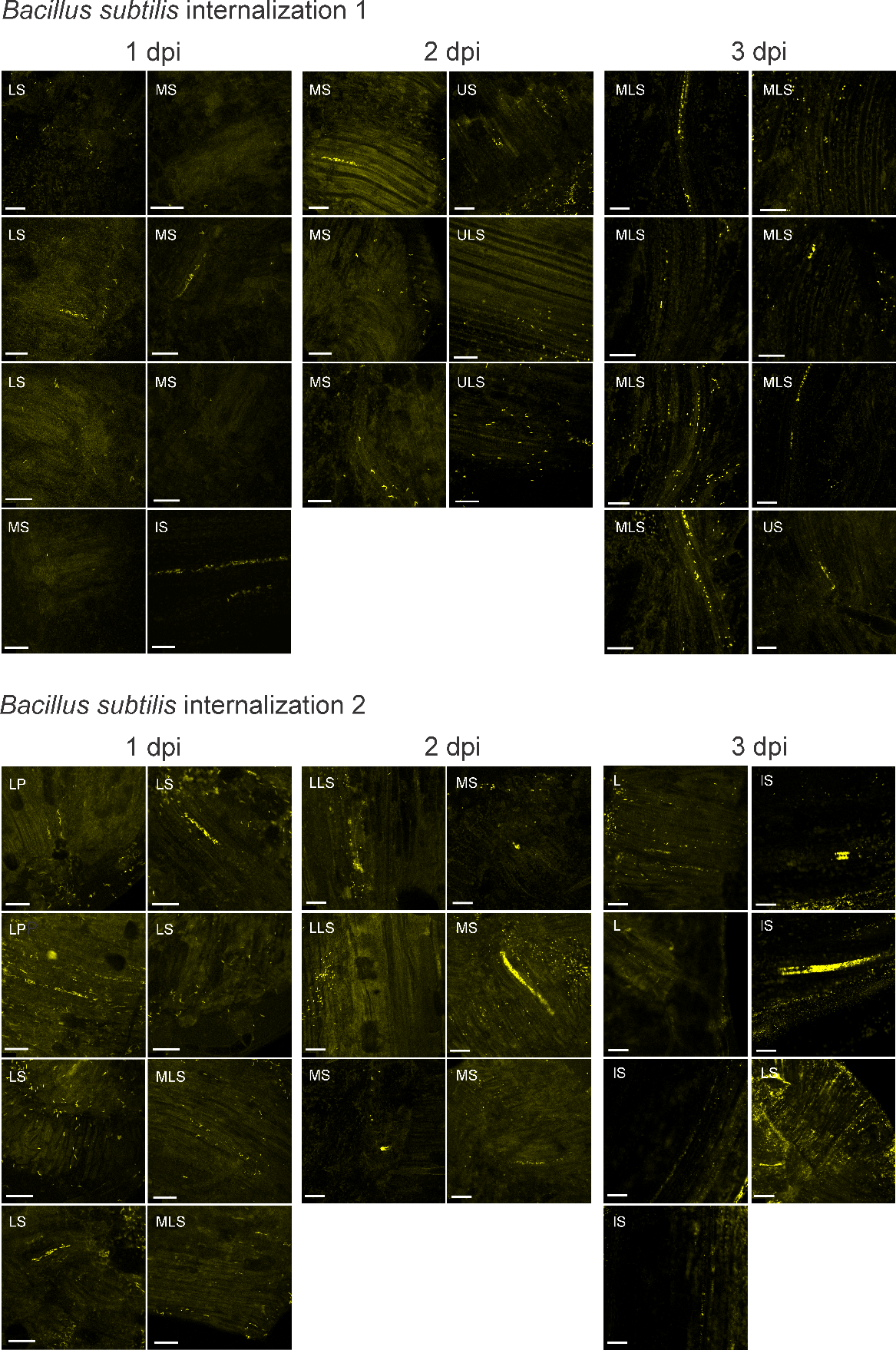


**Fig. S7: Observation of *B. subtilis* cells in potato stems and leaves after internalization.** Cells in potato cv. Rywal tissue were imaged in two different experiments by following YFP fluorescence up to three days after inoculation (dpi) of the tissue culture plants with *B. subtilis* PS-218 YFP culture (see methods section Plant Inoculations). YFP fluorescence is colored as yellow. Scale is 50 µm. Microscopy lif files are available on Zenodo (<https://doi.org/10.5281/zenodo.18174134>). LS – lower stem, MS – middle stem, MLS – middle stem longitudinal section, US – upper stem, ULS – upper stem longitudinal section, LP – leaf petiole, LLS – lower stem longitudinal section, L – leaf, lS – stem longitudinal section


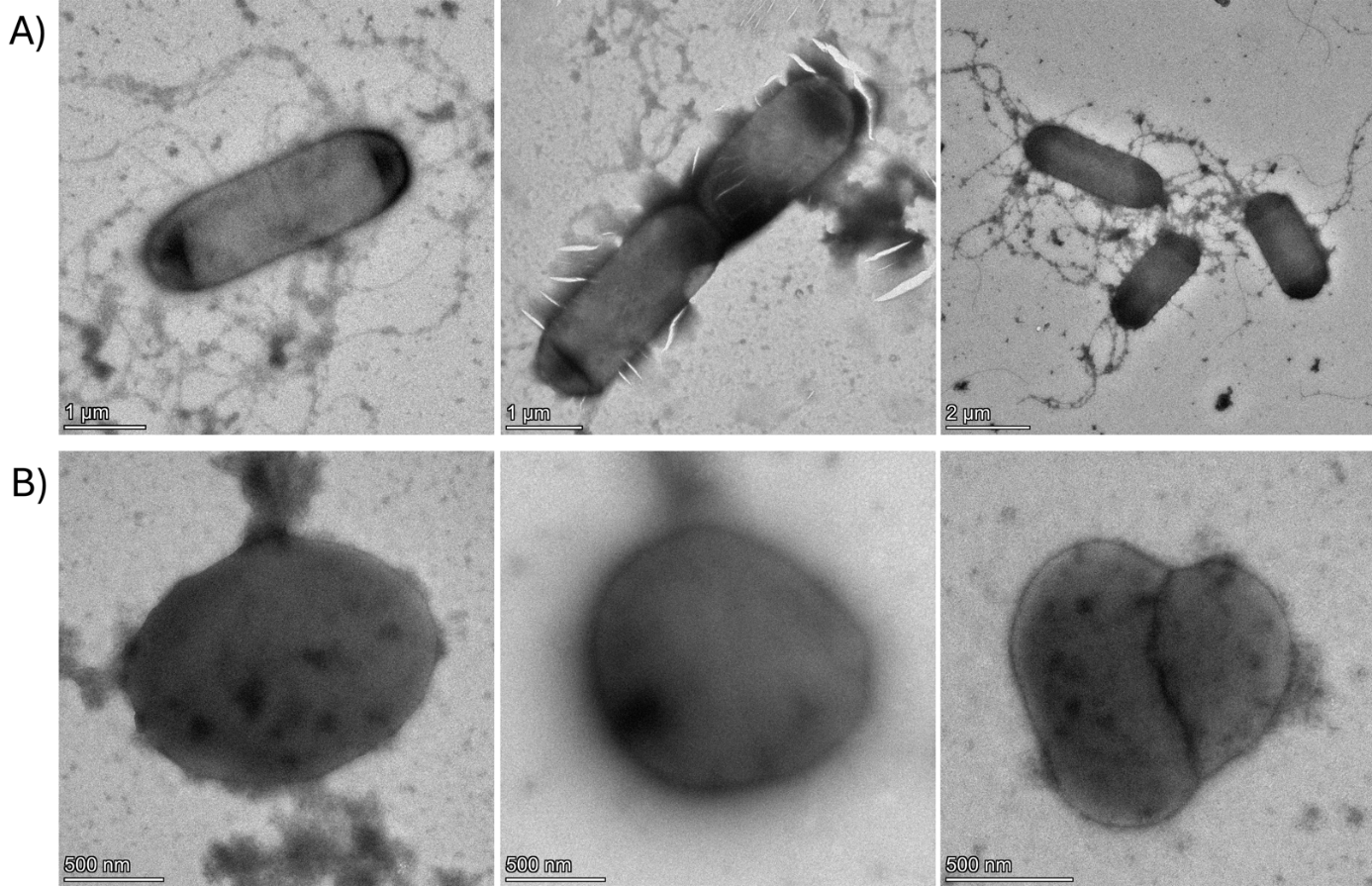


**(a)**

**(b)**

**Fig. S8: Morphology of *B. subtilis* cells in liquid culture (a) and potato leaf (b).** Potato cv. Rywal leaves were infiltrated with *B. subtilis* PS-216 YFP (10^7^ CFU/ml) and samples were collected for transmission electron microscopy three days after infiltration (b). Free-living bacteria in the liquid culture used for leaf inoculation were used as a control (a). Measure of scale is given on each figure. Additional images are available on Zenodo (<https://doi.org/10.5281/zenodo.18174134>).


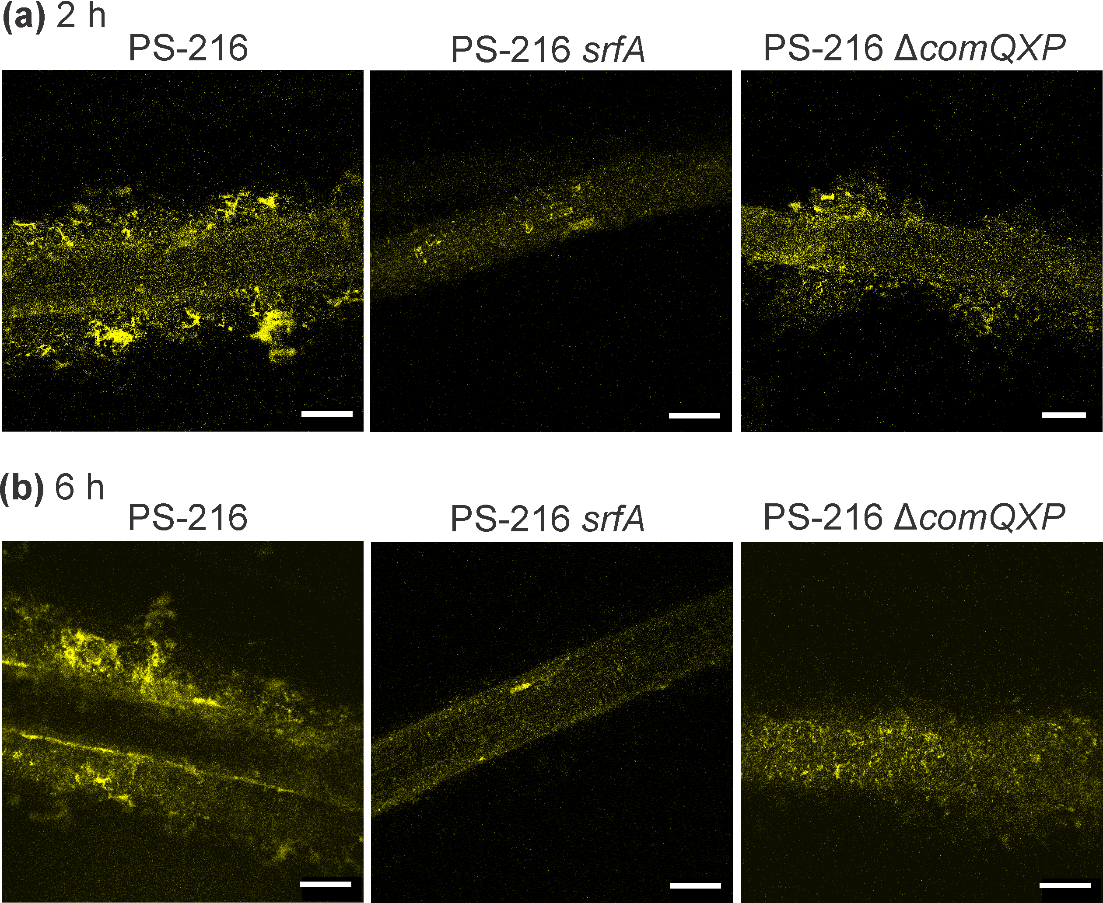


**Fig. S9: Biofilm formation on potato roots inoculated with *B. subtilis* mutants that have attenuated surfactin production.** Biofilm formation was imaged on potato roots of cv. Rywal by confocal microscopy after (a) 2-h and (b) 6-h incubation in YFP-labeled *B. subtilis* PS-216, *B. subtilis* PS-216 *srfA* and PS-216 *ΔcomQXP* culture (10⁸-10⁹ CFU/ml). YFP fluorescence is shown. Scale is 250 µm.


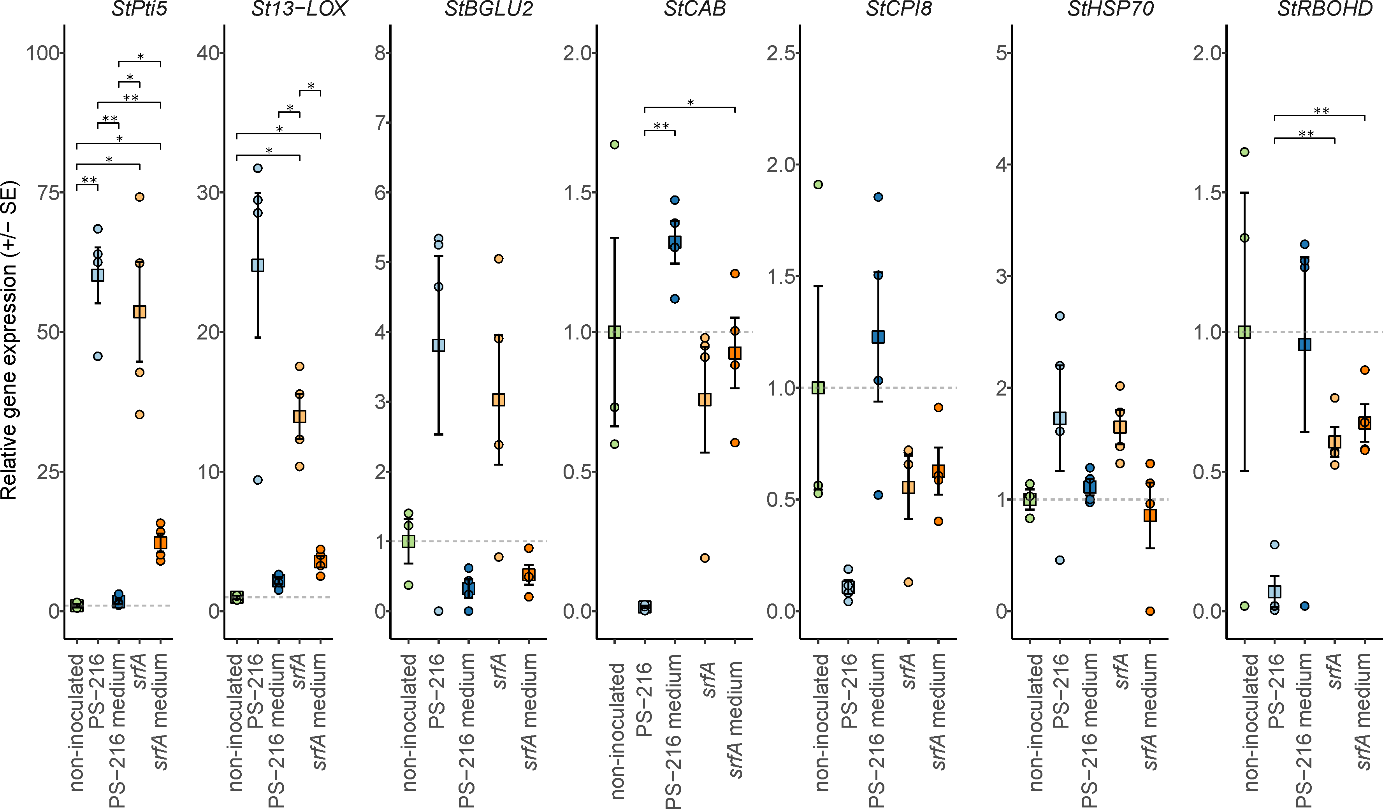


**Fig. S10: *B. subtilis*-produced secondary metabolites in the medium do not induce the same response in potato roots as inoculation with live bacteria.** Potato plants of cv. Rywal from tissue cultures were added to bacteria culture (YFP-labeled *B. subtilis* PS-216 and *B. subtilis* mutant PS-216 *srfA* with attenuated surfactin production) with OD_600_ 0.02 and incubated overnight. After 17 h, plants and bacteria were discarded and new plants were added to the conditioned medium and incubated for 26 h. As a positive control, plants were inoculated with YFP-labeled *B. subtilis* PS-216 and *B. subtilis* mutant PS-216 *srfA* with attenuated surfactin production as in previous experiments. As a negative control, non-inoculated samples (inoculated with MS30 medium) were used. Relative expressions (relative to the endogenous control, see methods) of several genes (*StPti5*, *St13-LOX*, *StBGLU2*, *StCAB*, *StCPI8*, *StHSP70*, *StRBOHD*) were measured in roots by quantitative PCR (qPCR). Games Howell post-hoc test was used to determine differences between groups (n = 4). For visualization purpose, relative gene expression was scaled to the average gene expression of non-inoculated group. Individual measurements (circles), mean (squares) and standard error are shown. Asterisks (*) denote statistically significant difference (p-value < 0.05). See Table S12 for experimental data.


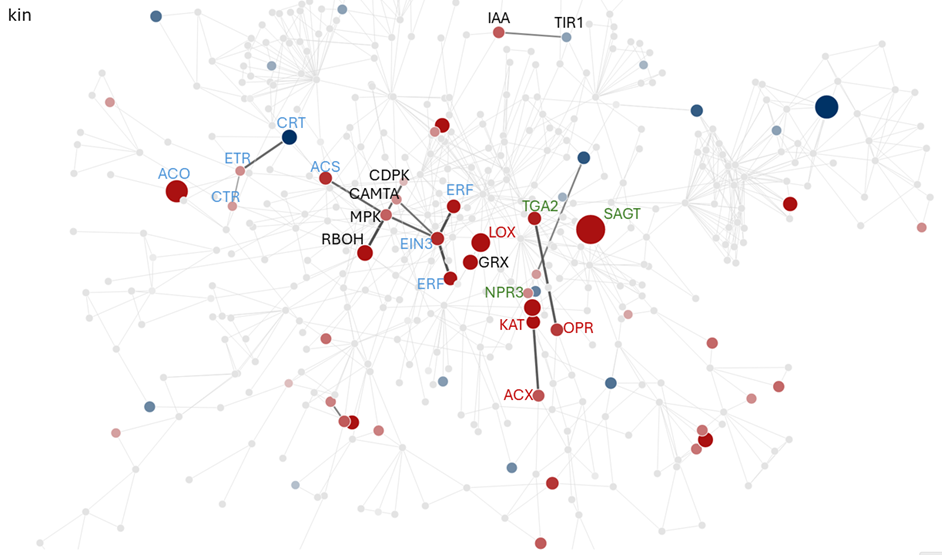


non-kin

isogenic


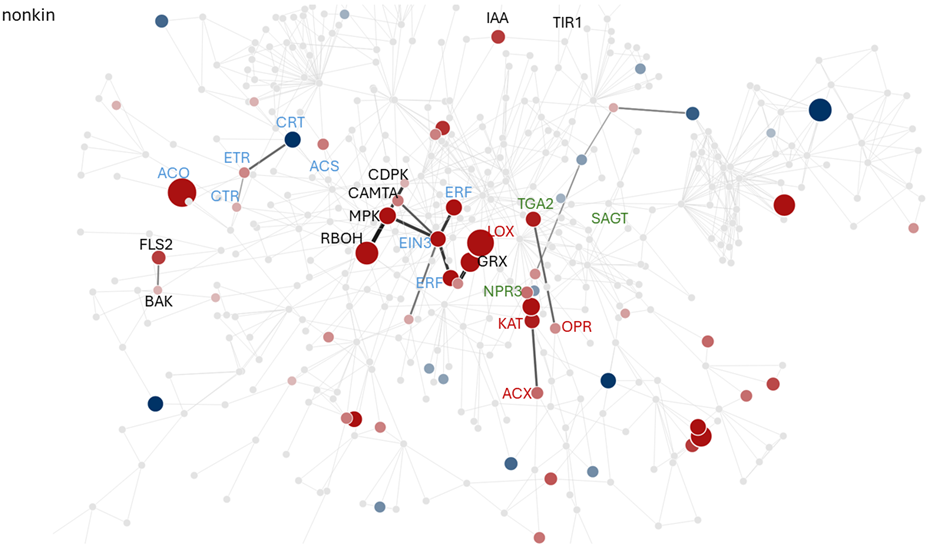


**Fig. S11: Regulated central signaling modules in potato roots in isogenic and non-kin interactions.** Isogenic: PS-218 YFP + PS-218 mKate2, non-kin: PS-216 YFP + PS-218 mKate2. RNA-seq data was overlayed with plant stress signaling prior knowledge network, built from experimental data on protein-protein interactions, protein-DNA interactions, and metabolic pathways (skm.nib.si) (Bleker et al., 2024) to determine regulated central signaling modules in isogenic and non-kin interactions. Genes from ethylene (blue), JA (red), SA (green) and other (black) signaling modules are shown. See Table S14 for the names and descriptions of the regulated genes.


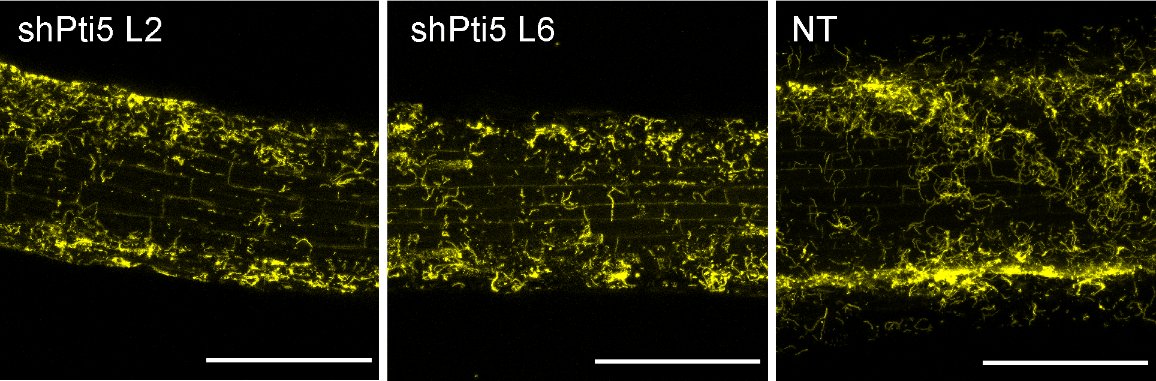


**Fig. S12: *B. subtilis* root colonization of transgenic potato cv. Rywal lines with silenced *StPti5* (shPti5 L2 and L6) and non-transgenic (NT) potato plant.** Plants were added to *B. subtilis* PS-218 YFP culture of high density (10^8^-10^9^ CFU/mL), incubated for 2 h and imaged under confocal microscope. YFP fluorescence is colored as yellow. Scale is 250 µm. Additional images are available on Zenodo (<https://doi.org/10.5281/zenodo.18174134>).


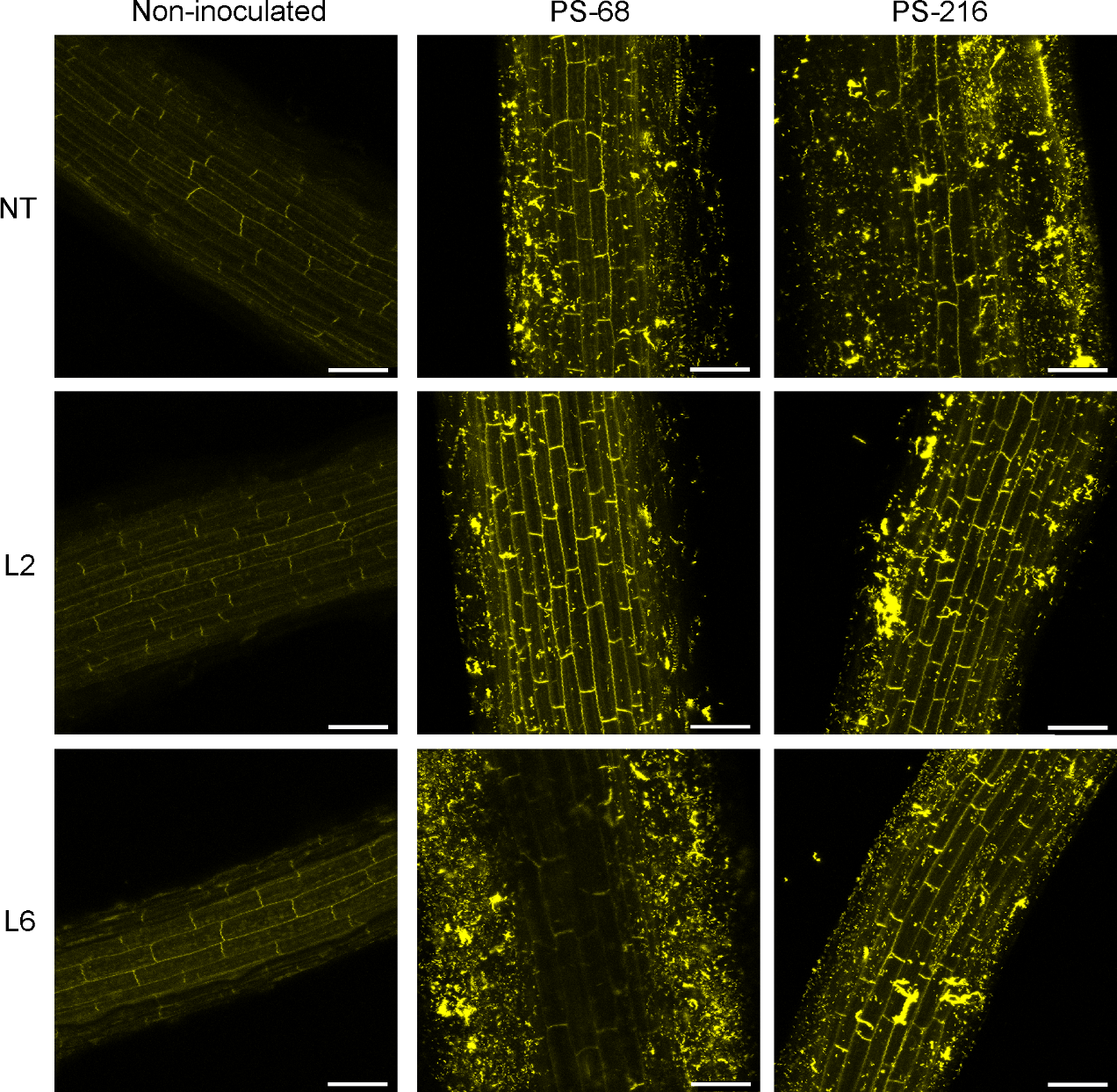


**Fig. S13: Biofilm formation on transgenic potato lines with silenced StPti5 (shPti5 L2 and shPti5 L6) and non-transgenic (NT) potato roots inoculated with two different *B. subtilis* strains.** Potato plants were added to *B. subtilis* PS-68 YFP or PS-216 YFP culture of high density (10^8^ - 10^9^CFU/mL), incubated for 2 h and imaged under confocal microscope. YFP fluorescence is colored as yellow. Scale is 100 µm. Additional images are available on Zenodo (<https://doi.org/10.5281/zenodo.18174134>).


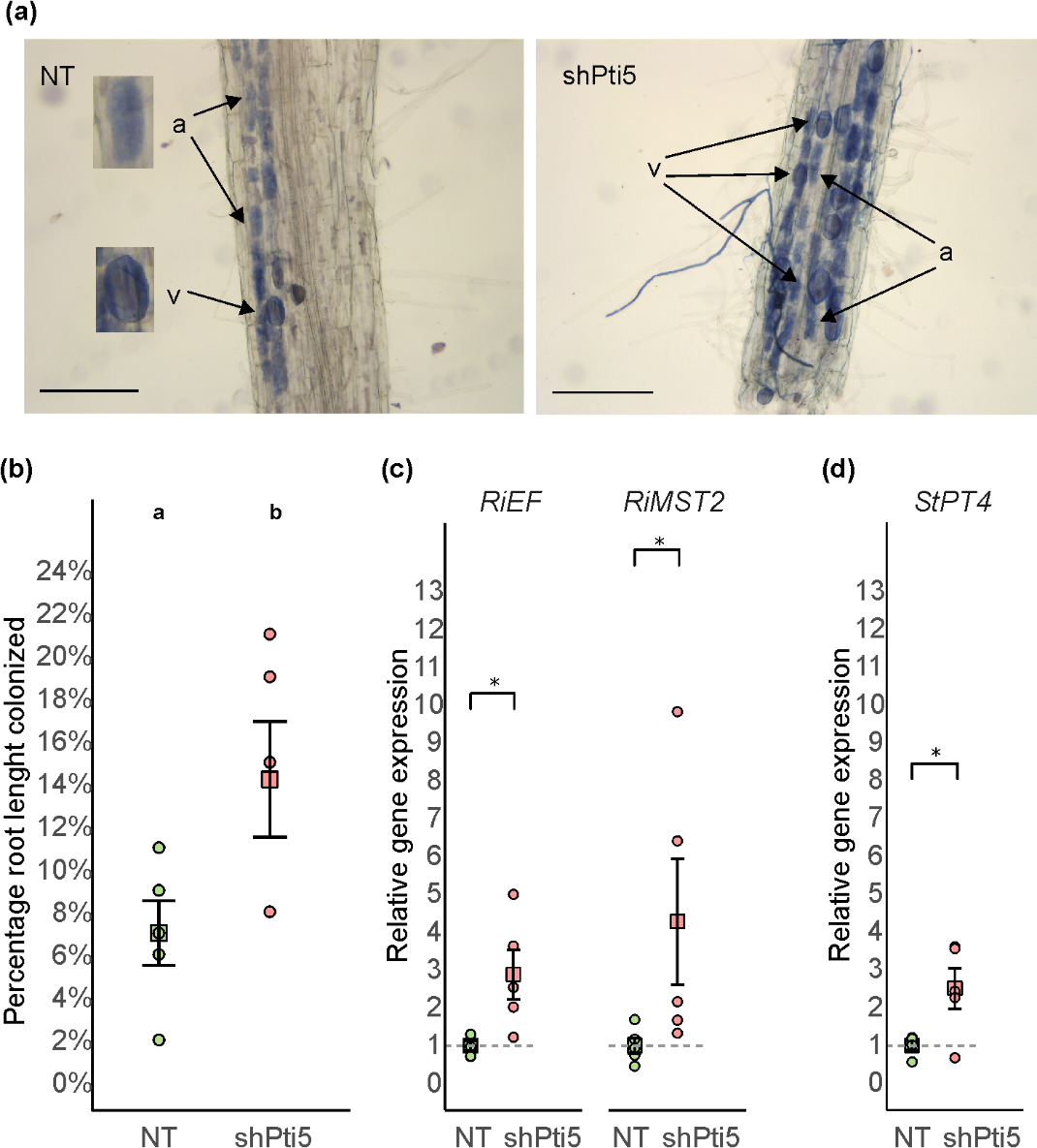


**Fig. S14: Mycorrhizal colonization of potato roots in non-transgenic potato plant (NT) and Pti5-silenced genotype line 2 (shPti5).** (a) Representative images of mycorrhizal colonization in the different genotypes. Fungal structures within the root cortex of potato plants are stained in blue (a: arbuscules, v: vesicles). Scale is 200 µm. (b) Percentage of root length colonized by the arbuscular mycorrhizal fungi *R. irregularis.* Percentage outcomes (n = 5) were analyzed using beta regression, with estimated marginal means (EMMs) computed for each genotype. Pairwise comparisons to the control group were conducted using Dunnett's method (dunnettx) in one-vs-control design. (c) Expression analysis of *R. irregularis* constitutive gene (RiEF) and symbiosis marker genes, the *R. irregularis* monosacaride transporter (RiMST2) and (d) the potato phosphate transporter (StPT4). For visualization purpose, relative gene expression was scaled to the average gene expression of control group (NT). Individual measurements (circles), mean (squares) and standard error are shown.

**Supporting Methods**

**Methods S1: Plant growth conditions**

Plantlets were grown in stem node tissue culture for two weeks after in-vitro micropropagation under controlled environmental conditions (22 ± 2 °C in the light and 19 ± 2 °C in the dark with 70–90 μmol m-2 s-2 radiation (OSRAM L 58 W/77 FLUORA lamps, Germany) and a 16-h photoperiod). When planted in soil, the plants were grown under environmental conditions 21 ± 2 °C in the light and 18 ± 1 °C in the dark, at a relative humidity of 75 % ± 2 %, with 90 µmol m-2 s-2 radiation (L36W/77 lamp, Osram, Germany) and a 16-h photoperiod (Baebler et al., 2009).

**Methods S2: *B. subtilis* strains**

Wild-type (WT) *B. subtilis* strains PS-216, PS-218 and PS-68 (Stefanic & Mandic-Mulec, 2009) tagged with red fluorescent protein (mKate2) or yellow fluorescent protein (YFP) gene linked to a constitutive hyperspank promoter were used, namely PS-218 amyE::Phypercl03-YFP (Sp) (PS-218 YFP), PS-216 amyE::Phypercl03-YFP (Sp) (PS-216 YFP), PS-68 amyE::Phypercl03-YFP (Sp) (PS-68 YFP), PS-218 amyE::Phyperspank-mKate2 (Chl) (PS-218 mKate2) and PS-216 amyE::Phyperspank-mKate2 (Chl) (PS-216 mKate2) (Stefanic et al., 2015; Kraigher et al., 2022). PS-216 mutants with impaired surfactin production PS-216 amyE::PhypercI03-YFP (Sp) srfA::Tn917 (mls) (PS-216 srfA YFP) (Kraigher et al., 2022) and QS mutant PS-216 ΔcomQXP::kan amyE::PhypercI03-YFP (Sp) (PS-216 ΔcomQXP YFP) (this work) were used. QS mutant PS-216 ΔcomQXP::kan amyE::PhypercI03-YFP (Sp) was constructed by transformation of PS-216 amyE::PhypercI03-YFP (Sp) (Kraigher et al., 2022) with plasmid pED302 (Tortosa et al., 2001), carrying a kanamycin cassette between degQ and comA, resulting in a deletion of the comQXP locus.

**Methods S3: *B. subtilis* IAA production detection**

Fresh bacterial cultures were prepared from single colonies in LB medium supplemented with 0.1 % tryptophan and incubated in the dark at 30 °C with shaking at 120 rpm for 48 h. After incubation, cultures were centrifuged at 16,000 g for 5 min, and the supernatant was transferred to a microtiter plate in triplicates. Equal volumes of Salkowski reagent (prepared by adding 1 ml of 0.5 M ferric chloride to 50 ml of 35 % perchloric acid, mixing well, and storing in a brown bottle) were added to each well. The mixture was incubated in the dark at room temperature for 30 min. Blanks were prepared similarly, using only LB medium with 0,1 % tryptophan. Absorbance was measured spectrophotometrically at 536 nm, and IAA concentrations were calculated using a standard curve prepared with IAA concentrations ranging from 10–100 µg/ml.

**Methods S4: Plant inoculations**

In initial experiments testing the plant's response and identify marker genes, two-week-old potato plants from tissue cultures were added to diluted bacterial cultures (OD_600_ = 0.02) in 12-well plates and incubated overnight before sampling.

To assess whether the conditioned media triggered any response in plants, plants were added to a diluted bacterial culture (10^7^ CFU/ml) and removed from the plate after incubation for 16 h at 11 rpm. The plant-conditioned medium was filtered through a 0.22 µm membrane to remove the bacterial cells. A new set of plants was added to the conditioned medium, incubated for 26 h, sampled, and stored at – 80 °C for qPCR analysis.

For studying *B. subtilis* spread through the plants, two-week-old potato plants, grown in tissue culture on solid MS30 medium, were inoculated with 300 µl of bacterial culture (OD_600_ = 0.3) and applied as drops onto the medium in close proximity to the roots. Roots, stems, leaves, and leaf petioles were sampled for observation under a confocal microscope 1-3 days post-inoculation (dpi). Internalization of bacteria in roots was observed after surface sterilization by incubation in 70 % ethanol for 1 min, 1 % sodium hypochlorite for 10 min and rinsed three times in autoclaved water. Stems, leaves, and leaf petioles were cut longitudinally and transversally to allow easier inspection of internal compartments.

When studying *B. subtilis* abundance in StPti5-silenced transgenic plants, on day four of bacterial growth, plants were incubated with bacteria (10^8^-10^9^ CFU/ml) for 2 h and then planted in soil (Fig. S1d-e). Plant shoots were dissected to yield approximately 20 mg of leaf tissue and 10 mg of apical tissue, and frozen for further analysis.

To study the expression of StPti5 downstream target genes, StPti5-silenced NahG (Coll et al., 2024) and NahG-Rywal plants were inoculated with potato virus Y^N-Wilga^ (PVY^N–Wi^; accession no. EF558545) or mock-inoculated, as described in Baebler et al. (2009).

**Methods S5: Mycorrhizal symbiosis establishment and quantification**

The mycorrhizal inoculum was maintained as a monoxenic culture, as *in vitro* culture of transformed carrot roots colonized by the arbuscular mycorrhizal fungus *Rhizophagus irregularis* (MUCL 57021, REKA Group. B.V., Bleiswijk, The Netherlands), produced and maintained in Gel-Gro (ICN Biochemicals, Aurora, OH, USA) as previously described in Chabot et al. (1992). Potato plantlets growing in 125 ml pots with a mixture of soil, sand, and vermiculite (3:2:1, v:v:v) were inoculated by adding a 1 cm piece of the monoxenic culture, containing approximately 50 *R. irregularis* spores, fungal hyphae, and colonized carrot roots. For the non-mycorrhizal controls, a piece of medium containing only uninfected carrot roots was applied. Plants were grown in a growth chamber (day/night cycle: 16 h, 24 °C/8 h, 19 °C; relative humidity: 50 %). The experiment was repeated twice, and eight independent plants per genotype were analyzed.

Mycorrhizal colonization of roots and establishment of symbiosis were evaluated histochemically and molecularly at 4 weeks post-inoculation. Mycorrhizal structures within the roots were observed and quantified after clearing the roots in 10 % KOH and staining the fungal structures with 5 % black ink in 2 % acetic acid solution, as described in García et al. (2020). The percentage of mycorrhizal colonization of roots was determined following the gridline intersection method (Giovannetti & Mosse, 1980) using a Nikon SMZ1000 stereomicroscope. To quantify the total amount of fungus within the root and assess symbiotic functionality, marker gene expression levels were analyzed by qPCR.

**Methods S6: Confocal microscopy settings**

The emission of YFP was followed after excitation with a 488 nm laser in the 505–560 nm range. The emission of mKate2 was followed after excitation with a 589 nm laser in the 605–650 nm range. Regions of interest (ROI) were scanned unidirectionally with a scan speed of 400 Hz and a frame average of at least 2. The images were processed to obtain maximum projections from Z-stacks for all channels and merged using Leica LAS X software (Leica Microsystems). The mean fluorescence intensity on confocal images was determined in LAS X software (Leica). For the laser power, PIN opening, and detector gain, please check metadata available on Zenodo (https://doi.org/10.5281/zenodo.18174134).

**Methods S7: Transmission electron microscopy sample preparation**

The infiltrated leaves were homogenized by chopping in 0.7 % NaCl solution, incubated for 1 hour on a low-speed shaker at 11 rpm, and the homogenate was passed through a 40 µm strainer. Separately, the bacterial culture used for infiltration was collected. Pellets of centrifuged liquid culture or leaf homogenate, infiltrated with *B. subtilis,* were resuspended in 0.1 M phosphate buffer with 2 % polyvinylpyrrolidone. Six µl of resuspension was applied to copper grids with or without glow discharge (400 mesh, formvar-carbon coated) for 5 min. Afterwards, the remaining resuspension was dabbed with filter paper. This procedure was repeated two more times. Afterwards, the grids were washed with bidistilled water and stained with one droplet of 1 % (w/v) water solution of uranyl acetate. Two grids were prepared for each sample.

**Methods S8: Nucleic acid isolation and purification**For qPCR, samples were homogenized in lysis buffer using FastPrep tubes and a FastPrep-24 homogenizer (MP Biomedicals). RNA was isolated from shoots using the RNeasy Plant Mini Kit (Qiagen) and from roots using RNeasy Plant Mini Kit (Qiagen) or RNeasy Micro Kit (Qiagen), according to the manufacturer's protocols. Concentration and purity of DNase-treated (0.5 μl of DNase I per μg RNA; Qiagen) total RNA were evaluated using the NanoDrop ND1000 spectrophotometer (Nanodrop technologies) and agarose gel electrophoresis and then reverse-transcribed using the High-Capacity cDNA Reverse Transcription Kit (Thermo Fisher Scientific) or High-Capacity RNA-to-cDNA Kit (Thermo Fisher Scientific). To determine *B. subtilis* abundance in systemic shoot tissue, samples were homogenized in lysis buffer using FastPrep tubes and a FastPrep-24 homogenizer (MP Biomedicals), and genomic DNA was isolated by MagMAX™ Plant DNA Isolation Kit (Thermo Fisher Scientific) according to the manufacturer's protocol. Concentration and purity were evaluated as stated above.

For RNA-Seq analysis of whole shoots and root elongation zones, samples were placed in FastPrep tubes (MP Biomedicals) and snap-frozen in liquid nitrogen. Samples were homogenized in lysis buffer using a FastPrep-24 homogenizer (MP Biomedicals). RNA was isolated using the RNeasy Plant Mini Kit (Qiagen) and treated with RNA Clean & Concentrator-5 Kit with DNase included (Zymo Research) according to the manufacturer’s protocols.

For RNA-Seq analysis to identify StPti5 downstream targets, 20 tissue sections encompassing the lesions were sampled from PVY^N-Wi^-inoculated leaves at 4 dpi and pooled. 20 tissue sections from mock-inoculated plants were pooled separately. Three pools were collected for each group. The pools were stored in 100 µl of RNAlater RNA Stabilization Solution (Thermo Fisher Scientific). RNA was extracted with TRIzol (Invitrogen) and Direct-zol RNA MicroPrep Kit, DNAse treated, and purified with RNA Clean & Concentrator kit (both Zymo Research) according to the manufacturer’s instructions with prior homogenization using TissueLyser (Qiagen).

**Methods S9: Statistical analysis of qPCR, microbial abundance and imaging data**

Statistical analysis of qPCR and microbial data was conducted in R version 4.4.1 (R Core Team, 2024).

To compare gene expression between two groups in qPCR data, effect sizes were estimated using the Wilcoxon method via the rstatix package v.0.7.2 (Kassambara, 2023), providing a robust measure of group differences. Statistical significance was assessed using a permutational t-test implemented via the MKinfer package v.1.2 (Kohl, 2024), which accommodates non-normal distributions and unequal variances. For qPCR data quantified by ΔΔCt method, pairwise comparisons between genotypes were conducted using exact permutation tests via the exactRankTests package v. 0.8-35 (Hothorn & Hornik, 2022). To account for multiple comparisons across genes, p-values were adjusted using the Benjamini-Hochberg (BH) procedure.

For comparisons involving multiple groups in qPCR data, the Games-Howell post-hoc test was applied using the rstatix package v.0.7.2 to assess pairwise differences in gene expression. For visualization, expression values were scaled to the average of the reference group to enable intuitive comparison across genes and conditions. All plots were generated using the ggplot2 package v.4.0.0 (Wickham, 2016).

For genotype-level comparisons, genotype was included as a fixed effect and root nested within genotype as a random effect; similarly, strain and plant were modelled for strain-level effects (Bates et al., 2015). Estimated marginal means (EMMs) and pairwise contrasts were computed using the emmeans package v.1.11.2-8 (Lenth *et al.*, 2025), with p-values adjusted for multiple testing using the BH method via the multcomp package v.1.4-28 (Hothorn *et al.*, 2008). Model fit and variance components were assessed using the MuMIn package v.1.48.11 (Barton, 2025), and results were visualized using ggplot2 v.4.0.0, including diagnostic plots and compact letter displays.

To assess *B. subtilis* abundance (coded as binary 0/1 outcomes) in shPti5 versus non-transgenic potato plants across different shoot tissues, one-sided Fisher’s Exact test for count data was applied.

To analyze the percentage of root length colonized by AMF across potato genotypes, beta regression was applied. Genotype was included as a predictor, and overall effects were assessed using joint tests. EMMs were computed using the emmeans package v.1.11.2-8. Pairwise comparisons were performed using Dunnett’s adjusted method (dunnettx), implemented via the multcomp package v.1.4-28 to generate compact letter displays for one-vs-control contrasts.

**Methods S10: RNA-Seq analysis**

For the RNA-seq experiment ‘Monocultures’, potato roots were inoculated with the culture of *B. subtilis* strains PS-216 YFP or PS-218 YFP. Plant material was sampled from roots after 2 and 26 h of incubation and from shoots after 26 h of incubation. For the RNA-seq experiment ‘Interactions’, potato roots were inoculated with individual *B. subtilis* strains (PS-216 YFP, PS-218 YFP, PS-218 mKate2), an isogenic mixture of strains (PS-218 mKate2 + PS-218 YFP), and a non-kin mixture of strains (PS-218 mKate2 + PS-216 YFP) and sampled after 2 h of incubation.

For both experiments, non-inoculated plants (incubated in MS30 medium without *B. subtilis*) were sampled as a control, and four biological replicates (plants) were analyzed for each treatment, including controls. Whole shoots and root elongation zones were sampled. RNA was isolated and purified as described above. RNA-seq strand-specific polyA selection-based library prep and sequencing on an Illumina NovaSeq 6000 were performed by Novogene (Germany), producing approximately 40 million 150-bp paired-end Illumina reads per sample. Sequencing quality control was performed using FastQC v0.11.9 (Andrews, 2010). Raw reads were adapter-trimmed and reads below average Phred base calling quality score 20 were removed using Trim Galore v0.6.10 (Krueger, 2021). Taxonomic classification of reads was performed using Centrifuge v1.0.4 (Kim *et al.*, 2016) in conjunction with the 2018 nt database. Reads were mapped to *Solanum tuberosum* Phureja clone DM 1-3 genome assembly v4.04 with STAR v2.7.5c (Dobin *et al.*, 2013) using the merged genome annotations (Petek *et al.*, 2020). Only uniquely mapped fragments were counted (--outFilterMultimapNmax 1) and additional mapping parameters were set (--outFilterMismatchNoverReadLmax 0.02, --quantTranscriptomeBan Singleend, --outFilterType BySJout, --alignSJoverhangMin 10, --alignSJDBoverhangMin 1, --alignIntronMin 20, --alignIntronMax 10000, --alignMatesGapMax 10000). Centrifuge and mapping results showed substantial contamination of sample BS472, therefore we excluded this sample from further analysis.

For RNA-Seq analysis to identify StPti5 downstream targets, 20 tissue sections encompassing the lesions were sampled from PVY^N-Wi^-inoculated leaves at 4 dpi and pooled. 20 tissue sections from mock-inoculated plants were pooled separately. Three pools were collected for each group. RNA was isolated and purified as described above. Libraries were prepared using SMART-Seq_v4 Ultra Low Input RNA Kit (TaKaRa) and Nextera XT kit (Illumina) at LC Sciences, and sequenced on the HiSeq4000 platform (Illumina) using 150-bp paired-end reads. Sequencing quality control was performed using FastQC v0.12.1 (Andrews, 2010). Raw reads were adapter-trimmed and reads below average Phred base calling quality score 20 were removed using cutadapt v4.4 (Martin, 2011). Reads were mapped to *Solanum tuberosum* Phureja clone DM 1-3 genome assembly v4.04 with STAR 2.7.8a (Dobin *et al.*, 2013) using the merged genome annotations (Petek *et al.*, 2020) with the following parameters: (--outFilterMultimapNmax 10 --outFilterMismatchNoverReadLmax 0.05, --quantTranscriptomeBan Singleend, --outFilterType BySJout, --alignSJoverhangMin 10, --alignSJDBoverhangMin 1, --alignIntronMin 20, --alignIntronMax 10000, --alignMatesGapMax 10000).

Differential gene expression analysis was performed in R v3.6.1 using the *limma* package v3.40.6 (Ritchie *et al.*, 2015). Low-expressed genes were filtered by retaining only genes with raw read counts above 100 in at least 11 samples for RNA-seq experiment ‘Interactions’, above 50 counts in at least 4 samples for RNA-seq experiment ‘Monocultures’, and above 50 counts in at least 3 samples for RNA-Seq experiment ‘StPti5 downstream target identification’. Root and shoot differential expression analysis was performed separately in the experiment ‘Monocultures’. Normalized counts were transformed using voom function, followed by linear model fit, and statistics calculation for defined contrasts using the eBayes function.

Gene Set Enrichment Analysis was performed with the GSEA Desktop application v4.1.0 using the MapMan ontology-based gene sets and non-filtered normalized read counts, comparing different *Bacillus* treatments to non-inoculated plants to identify significantly altered regulation of processes and functionally related gene groups (FDR corrected q-value < 0.10). Results of all GSEA comparisons were merged using R v4.3.1 (R Core Team, 2023). GSEA figure was generated using the adjusted gseaFromStats function from biokit v0.1.1 package (Rodríguez-Córdoba, 2023).

**Methods S11: DAP-seq**

25 ml of *E. coli* cultures carrying MBP-Pti5 construct were induced with 1 mM isopropyl β-D-1-thiogalactopyranoside (IPTG) at 18 °C overnight. Cells were then pelleted and kept frozen at -80 °C until use. Genomic DNA (gDNA) extracted from *S. tuberosum* cv. Désirée was fragmented in a Covaris M220 ultrasonicator using the 200 bp target peak protocol to generate Illumina-compatible libraries. The DNA Affinity Purification (DAP) assay was performed as described previously (Bartlett *et al.*, 2017; Franco-Zorrilla & Prat, 2021). Cleared soluble protein extracts were obtained from frozen *Escherichia coli* pellets, and 400 µl were incubated with 25 µl of pre-washed amylose magnetic beads (New England Biolabs), washed with PBS, and finally incubated with 200 ng of the gDNA library. The bound DNA was recovered and amplified using indexed Illumina-compatible primers through 20 cycles of PCR. The input samples were a result of direct amplification of the libraries through 11 cycles of PCR. PCR products were purified using AMPure XP beads (Beckman Coulter), indexed libraries mixed into a single pool, and sequenced on a NovaSeq (150 bp paired-end) by Novogene. Raw reads in FASTQ format were trimmed for adaptor sequences, and low-quality reads filtered (Phred < 20) with Trim Galore v0.6.10 (Krueger *et al.*, 2023). Reads were mapped with Bowtie2 (v2.5.2) (Langmead & Salzberg, 2012) to the *S. tuberosum* DM v4.03 genome version (The Potato Genome Sequencing Consortium, 2011) using default parameters. For visualization in IGB v9.1.10, BAM files were converted to binary BIGWIG interval files with the ‘BAM coverage’ tool in deepTools2 v3.5.4 (Ramírez *et al.*, 2016), using a bin size of 10 bases and normalized to bins per million. Peak calling was performed with GEM v3.4 (Guo *et al.*, 2012), using the corresponding ‘Input’ sample as negative control with the parameters ‘--range 200 --smooth 0 --mrc 1 --fold 2 --q 1.301029996 --k_min 6 --k_max 20 - k_seqs 600 --k_neg_dinu_shuffle --pp_nmotifs 1’. Two technical replicates were performed, and a list of high-confidence peaks detected in both replicates was generated with BEDTOOLS v2.30.0 bedintersect (Quinlan, 2014). Significant StPti5-bound peaks were annotated relative to the nearest gene with the R library ChIPSeeker (Yu *et al.*, 2015). BEDTOOLS getfasta (Quinlan, 2014) was used to obtain 200-bp sequences from the enriched peaks. The top-ranking 1000 sequences were used for the discovery of consensus motifs with MEME v5.5.5 with the command ‘meme-mod zoops -minw 8 -maxw 15 -meme-nmotifs 3’ and a background of Markov model 0 (Bailey *et al.*, 2009).

**Supporting references**

**Andrews S. 2010.** *FastQC: A Quality Control Tool for High Throughput Sequence Data.* GitHub. URL <https://github.com/s-andrews/FastQC>

**Baebler Š, Krečič-Stres H, Rotter A, KogovŠek P, Cankar K, Kok EJ, Gruden K, KovaČ M, Žel J, Pompe-Novak M, *et al.* 2009.** PVY^NTN^ elicits a diverse gene expression response in different potato genotypes in the first 12 h after inoculation. *Molecular Plant Pathology* **10**: 263–275.

**Bailey TL, Boden M, Buske FA, Frith M, Grant CE, Clementi L, Ren J, Li WW, Noble WS. 2009.** MEME Suite: Tools for motif discovery and searching. *Nucleic Acids Research* **37**.

**Bartlett A, O’Malley RC, Huang SSC, Galli M, Nery JR, Gallavotti A, Ecker JR. 2017.** Mapping genome-wide transcription-factor binding sites using DAP-seq. *Nature Protocols* **12**: 1659–1672.

**Barton K. 2025.** *MuMIn: Multi-Model Inference R package version 1.48.11.* CRAN. URL <https://cran.r-project.org/web/packages/MuMIn/index.html>

**Bates D, Mächler M, Bolker BM, Walker SC. 2015.** Fitting linear mixed-effects models using lme4. Journal of Statistical Software 67.

**Chabot S, Bécard G, Piché Y. 1992.** Life Cycle of Glomus Intraradix in Root Organ Culture. *Mycologia* **84**: 315–321.

**Dobin A, Davis CA, Schlesinger F, Drenkow J, Zaleski C, Jha S, Batut P, Chaisson M, Gingeras TR. 2013.** STAR: ultrafast universal RNA-seq aligner. *Bioinformatics* **29**: 15–21.

**Franco-Zorrilla JM, Prat S. 2021.** DAP-Seq Identification of Transcription Factor-Binding Sites in Potato. In: Dobnik D, Gruden K, Ramšak Ž, Coll A, eds. *Solanum tuberosum Methods in Molecular Biology.* Humana, New York, NY, 123–142.

**García JM, Pozo MJ, López-Ráez JA. 2020.** Histochemical and Molecular Quantification of Arbuscular Mycorrhiza Symbiosis. In: Rodríguez-Concepción M, Welsch R, eds. *Plant and Food Carotenoids.* Methods in Molecular Biology, Humana, New York, 293-299.

**Giovannetti M, Mosse B. 1980.** An evaluation of techniques for measuring vesicular arbuscular mycorrhizal infection in roots. *New Phytologist* **84**: 489–500.

**Guo Y, Mahony S, Gifford DK. 2012.** High Resolution Genome Wide Binding Event Finding and Motif Discovery Reveals Transcription Factor Spatial Binding Constraints. *PLoS Computational Biology* **8**.

**Hothorn T, Hornik K. 2022.** *exactRankTests: Exact Distributions for Rank and Permutation Tests R package version 0.8-35.* CRAN. URL <https://cran.r-project.org/web/packages/exactRankTests/index.html>

**Hothorn T, Bretz F, Westfall P. 2008.** Simultaneous inference in general parametric models. *Biometrical Journal* **50**: 346–363.

**Kassambara A. 2023.** *rstatix: Pipe-Friendly Framework for Basic Statistical Tests R package version 0.7.2.* CRAN. URL <https://cran.r-project.org/web/packages/exactRankTests/index.html>

**Kohl M. 2024.** *MKinfer: Inferential Statistics R package version 1.2.* CRAN. URL https://cran.r-project.org/web/packages/MKinfer/index.html

**Kim D, Song L, Breitwieser FP, Salzberg SL. 2016.** Centrifuge: rapid and sensitive classification of metagenomic sequences. *Genome Research* **26**: 1721–1729.

**Kraigher B, Butolen M, Stefanic P, Mandic Mulec I. 2022.** Kin discrimination drives territorial exclusion during *Bacillus subtilis* swarming and restrains exploitation of surfactin. *ISME Journal* **16**: 833–841.

**Krueger F. 2021.** *Trimgalore .* GitHub. URL <https://github.com/FelixKrueger/TrimGalore>

**Krueger F, James F, Ewels P, Afyounian E, Weinstein M, Schuster-Boeckler B, Hulselmans G, Sclamons. 2023.** *FelixKrueger/TrimGalore: v0.6.10 - add default decompression path (0.6.10).* Zenodo. URL <https://zenodo.org/records/7598955>

**Langmead B, Salzberg SL. 2012.** Fast gapped-read alignment with Bowtie 2. *Nature Methods* **9**: 357–359.

**Lenth R V., Piaskowski J, Banfai B, Bolker B, Buerkner P, Giné-Vázquez I, Hervé M, Jung M, Love J, Miguez F, *et al.* 2025.** *emmeans: Estimated Marginal Means, aka Least-Squares Means R package version 1.11.2-8.* CRAN. URL https://cran.r-project.org/web/packages/emmeans/

**Martin M. 2011.** Cutadapt removes adapter sequences from high-throughput sequencing reads. *EMBnet.journal*: 10–12.

**Petek M, Zagorščak M, Ramšak Ž, Sanders S, Tomaž Š, Tseng E, Zouine M, Coll A, Gruden K. 2020.** Cultivar-specific transcriptome and pan-transcriptome reconstruction of tetraploid potato. *Scientific Data* **7**.

**Quinlan AR. 2014.** BEDTools: The Swiss‐Army Tool for Genome Feature Analysis. *Current Protocols in Bioinformatics* **47**.

**R Core Team. 2023.** *R: A Language and Environment for Statistical Computing.* R Foundation for Statistical Computing, Vienna. URL http://www.r-project.org.

**Ramírez F, Ryan DP, Grüning B, Bhardwaj V, Kilpert F, Richter AS, Heyne S, Dündar F, Manke T. 2016.** deepTools2: a next generation web server for deep-sequencing data analysis. *Nucleic Acids Research* **44**: W160–W165.

**Ritchie ME, Phipson B, Wu D, Hu Y, Law CW, Shi W, Smyth GK. 2015.** Limma powers differential expression analyses for RNA-sequencing and microarray studies. *Nucleic Acids Research* **43**: e47.

**Rodríguez-Córdoba M. 2023.** *biokit: Toolkit for the automatic analysis of omics data.* github. URL https://martingarridorc.github.io/biokit/

**Stefanic P, Mandic-Mulec I. 2009.** Social interactions and distribution of *Bacillus subtilis* pherotypes at microscale. *Journal of Bacteriology* **191**: 1756–1764.

**Stefanic P, Kraigher B, Lyons NA, Kolter R, Mandic-Mulec I. 2015.** Kin discrimination between sympatric *Bacillus subtilis* isolates. *Proceedings of the National Academy of Sciences of the United States of America* **112**: 14042–14047.

**The Potato Genome Sequencing Consortium. 2011.** Genome sequence and analysis of the tuber crop potato. *Nature* **475**: 189–195.

**Tortosa P, Logsdon L, Kraigher B, Itoh Y, Mandic-Mulec I, Dubnau D. 2001.** Specificity and genetic polymorphism of the *Bacillus* competence quorum-sensing system. *Journal of Bacteriology* **183**: 451–460.

**Wickham H. 2016.** *ggplot2*. Cham: Springer International Publishing.

**Yu G, Wang LG, He QY. 2015.** ChIP seeker: An R/Bioconductor package for ChIP peak annotation, comparison and visualization. *Bioinformatics* **31**: 2382–2383.
